# X-THREAT framework for adaptive and explainable deep learning-based minority-class and zero-day cyber threat detection

**DOI:** 10.1038/s41598-026-63529-5

**Published:** 2026-09-15

**Authors:** Samina Naz Qaisarani, Sahar K. Badri, Asad Khattak, Daniyal M. Alghazzawi, Mohammed Yahya Alghamdi, Mona Alkhozae, Abeer Almakky, Rania M. Alhazmi, Muhammad Zubair Asghar

**Affiliations:** 1 Gomal Research Institute of Computing (GRIC), Faculty of Computing, Gomal University, D.I.Khan (KP), Pakistan; 2 Department of Information Systems, Faculty of Computing and Information Technology, King Abdulaziz University, Jeddah, Saudi Arabia; 3 College of Technological Innovation, Zayed University, P.O. Box 144534, Abu Dhabi, United Arab Emirates; 4 Department of Computer Science, Faculty of Computing and Information, Al-Baha University, Al Baha, Saudi Arabia; 5 Department of Information Technology, Faculty of Computing and Information Technology, King Abdulaziz University, Jeddah, Saudi Arabia

**Keywords:** Adaptive intrusion detection, Explainable AI (XAI), Hybrid CNN-BiLSTM, VAE-GAN synthesis, Minority-class threat detection, Zero-day attack detection, SHAP-based interpretability, Network traffic analysis, Deep learning security, Cyber threat simulation, Engineering, Mathematics and computing

## Abstract

The dynamic nature of cyber threats—particularly minority-class and zero-day attacks—poses significant challenges to existing intrusion detection systems (IDS), which often lack adaptability, interpretability, and robustness. This paper presents X-THREAT, an adaptive and explainable deep learning framework designed to improve detection of rare and previously unseen cyber threats. Unlike prior approaches that treat detection, augmentation, and explainability separately, X-THREAT integrates these components within a unified pipeline. A hybrid VAE–GAN module enhances minority-class representation during training, while a CNN–BiLSTM–attention model performs threat detection, and a context-aware SHAP mechanism provides interpretable insights into model decisions. Experimental results on three benchmark datasets—CICIDS2017, UNSW-NB15, and ToN-IoT—show that X-THREAT consistently outperforms representative deep learning baselines. The model achieves 96.3% accuracy and 95.5% F1-score on CICIDS2017, 94.1% accuracy and 92.5% F1-score on UNSW-NB15, and 93.2% accuracy and 91.5% F1-score on ToN-IoT, along with improved minority-class recall and reduced false positive rates. AUC-ROC values of 0.97, 0.95, and 0.94 further demonstrate strong discriminative capability. Zero-day detection capability is evaluated using a leave-one-attack-class-out protocol, and interpretability is assessed through fidelity analysis and comparison with static SHAP and LIME methods. Results indicate consistent performance across independently evaluated datasets, suggesting potential for generalization. From a deployment perspective, computationally intensive components are restricted to offline training, while inference relies on the detection model with optional explanation. However, latency, resource usage, and edge deployment feasibility were not explicitly benchmarked and remain important directions for future work. Overall, X-THREAT provides a robust and interpretable framework for modern intrusion detection, with improved performance on minority-class and zero-day threat scenarios.

**Supplementary Information:**

The online version contains supplementary material available at https://doi.org/10.1038/s41598-026-63529-5.

## Introduction

### Background

The rapid growth of cloud computing, Internet of Things (IoT) systems, and digital infrastructures has significantly expanded the attack surface of modern networks, making Intrusion Detection Systems (IDS) critical for identifying malicious activity. However, traditional signature-based and rule-driven IDS remain ineffective against stealthy and zero-day attacks that do not match known patterns^1^.

Deep learning (DL)-based IDS have improved the detection of complex and nonlinear traffic patterns, yet they still face key limitations, including limited interpretability, poor adaptability to evolving threats, and sensitivity to imbalanced data distributions^1^. Consequently, these systems often exhibit high false positive rates and reduced effectiveness in detecting minority-class and previously unseen attack patterns^2^.

These challenges highlight the need for IDS frameworks that are not only accurate but also adaptive and interpretable. While Explainable AI (XAI) has been introduced to enhance transparency, effective IDS solutions must additionally incorporate temporal context, adaptive learning, and improved exposure to rare and evolving threat patterns to remain operationally reliable^3^.

### Motivation

While deep learning (DL)-based intrusion detection systems (IDS) have improved detection accuracy, their practical deployment remains constrained by limited interpretability and reduced adaptability to evolving threats. Explainable AI (XAI) techniques such as SHAP and LIME enhance transparency; however, they are typically applied in static, post-hoc forms that do not capture temporal dependencies or contextual variations in cyber attack behavior, particularly under zero-day evaluation settings^1^.

In addition, most IDS frameworks do not adequately address minority-class imbalance, resulting in reduced sensitivity to rare but critical attacks and limited robustness when encountering previously unseen threat patterns^2^. The absence of integrated generative mechanisms further restricts exposure to diverse variations of known attack behaviors, limiting generalization capability.

To address these challenges, we propose X-THREAT, a unified framework that integrates temporal detection, generative augmentation, and context-aware explainability to improve robustness, interpretability, and minority-class detection performance.

### Research gap and baseline studies

Despite progress in applying deep learning (DL) and explainable artificial intelligence (XAI) to intrusion detection systems (IDS), important limitations remain in current approaches. First, many DL-based IDS lack adaptive temporal modeling and therefore struggle to generalize beyond observed attack patterns. For example, Samed and Sagiroglu^2^ incorporated SHAP and LIME into IDS pipelines, but their explanations remained static and did not account for evolving threat behavior. Second, several IDS frameworks, such as Mohale and Obagbuwa^3^, rely only on available labeled data and do not incorporate generative augmentation, limiting robustness under minority-class imbalance. Third, XAI-enabled IDS models, including L-XAIDS by Muhammad et al.^4^, typically employ single, context-independent explanation methods, reducing explanation fidelity and operational usefulness. These limitations highlight the need for an IDS framework that jointly supports adaptive temporal detection, generative augmentation for minority-class learning, and context-aware SHAP-based explainability.

### Problem statement

Despite improvements in detection accuracy and interpretability, existing IDS frameworks remain limited by:

i. insufficient adaptability to evolving attack patterns,
ii. static and context-insensitive explanation mechanisms, and.
iii. lack of integrated strategies for addressing minority-class imbalance.

Furthermore, prior approaches typically treat detection, augmentation, and explainability as independent components, resulting in fragmented pipelines that fail to capture their interdependencies.

To overcome these limitations, we propose X-THREAT, a unified framework that integrates temporal detection, VAE–GAN-based augmentation, and context-aware SHAP explainability within a single learning pipeline. Unlike prior works^2–4^, this tightly coupled design enables improved minority-class detection performance and more reliable interpretability under zero-day evaluation settings.

### Research questions (RQs)

Following research questions are posed.

- RQ1: How effectively does the X-THREAT framework detect known (minority-class) and zero-day attack types across benchmark datasets compared to standard deep learning baselines? This responds to the generalization and adaptability gap in Baseline Study^2^, where static modeling limited responsiveness to zero-day threats.
- RQ2: What is the impact of incorporating generative threat simulation on the detection of minority-class and zero-day attack patterns within the X-THREAT framework? Addresses Baseline Study^3^, which omitted synthetic threat generation, leading to low recall for minority-class events.
- RQ2: How does the integration of a VAE–GAN-based generative module affect the detection of minority-class attack patterns and generalization under zero-day evaluation settings?

RQ3: How does context-aware SHAP-based explainability, incorporating dynamic attribution reweighting, influence the interpretability and fidelity of detection decisions in X-THREAT compared to static explanation methods?

This directly addresses Baseline Study^4^, where reliance on static, single-modality XAI techniques limited interpretive depth and contextual adaptability. Table 1 summarizes the alignment between research questions, motivation, and identified research gaps.

**Table 1:** Mapping of research questions to motivations and research gaps.

Research question (RQ)	Motivation	Connection to research gap/baseline study
RQ1: How effectively does the X-THREAT framework detect known and zero-day attack types across benchmark datasets compared to standard deep learning baselines?	While existing DL-based IDS achieve high accuracy on known attacks, they struggle to generalize to zero-day threats and across datasets. A hybrid attention-based architecture may enhance adaptability to diverse traffic contexts.	Addresses the adaptability and generalization gap in Baseline Study^2^ (Samed and Sagiroglu), which lacked temporal modeling and failed to adapt to zero-day attack behavior. Aligns with the problem statement’s emphasis on robust, cross-scenario detection.
RQ2: What is the impact of incorporating generative threat simulation on the detection of minority-class and zero-day attack patterns within X-THREAT?	Most IDS are trained solely on historical attacks and lack proactive exposure to minority-class and zero-day threats. Generative modeling could improve recall for minority-class events and enhance robustness.	Directly responds to the deficiency in Baseline Study^3^ (Mohale and Obagbuwa), which omitted generative components, limiting resilience to zero-day attacks. Reinforces the call for synthetic augmentation in the problem formulation.
RQ3: How does context-aware SHAP-based explainability, incorporating dynamic attribution reweighting, influence the interpretability and fidelity of detection decisions in X-THREAT compared to static explanation methods?	Existing XAI integrations often rely on one-size-fits-all explanations that fail to capture contextual factors such as protocol behavior and temporal dynamics. Context-aware SHAP-based explainability, incorporating dynamic attribution reweighting, can enhance interpretive clarity by aligning feature attributions with operational network conditions.	Builds directly on the limitations of Baseline Study (Muhammad et al.^4^), where static, single-modality explanations were decoupled from model behavior. Aligns with the problem’s emphasis on contextual transparency and operational trust.

### Research contributions

This study presents X-THREAT, a unified intrusion detection framework that integrates adaptive detection, generative augmentation, and context-aware explainability to address limitations in adaptability, interpretability, and robustness identified in prior studies^2–4^. The key contributions are:

- *Adaptive Detection (RQ1)* A CNN–BiLSTM architecture with temporal attention that captures spatial and sequential patterns, enabling robust detection of known and minority-class threats under zero-day evaluation settings.
- *Generative Augmentation (RQ2)* A VAE–GAN module that learns structured representations of attack behavior and generates semantically consistent samples to enhance minority-class detection and feature space coverage.
- *Context-Aware Explainability (RQ3)* A SHAP-based mechanism with contextual attribution reweighting, providing consistent and interpretable explanations aligned with operational conditions.
- *Unified Evaluation Framework (RQ1–RQ3)* A comprehensive evaluation using CICIDS2017, UNSW-NB15, and ToN-IoT datasets, incorporating minority-class recall, false positive rate, and a strict attack-class holdout protocol for assessing generalization to unseen attacks.

#### Summary

These contributions collectively address key limitations in prior IDS approaches—namely static modeling^2^, lack of generative augmentation^3^, and context-insensitive explanations^4^—resulting in a more adaptive and interpretable intrusion detection framework.

### Key terms and definitions

This section summarizes key terms used in this study:

- *Minority-class threats* Known attack classes with very limited representation in the training data (typically < 0.5% of records). These classes remain available during training and are therefore rare but not unseen.
- *Zero-day threats* Attack classes that are completely excluded from training and validation and appear only during testing under the leave-one-attack-class-out protocol. In this study, zero-day threats are evaluated as unseen classes rather than generated synthetically.
- *Synthetic attacks* VAE–GAN-generated samples derived from the distributions of seen minority-class attacks. These samples are used only for training-time augmentation to mitigate class imbalance and improve representation learning. They are not treated as true zero-day attacks.
- *X-THREAT* A unified intrusion detection framework integrating CNN–BiLSTM-based detection (RQ1), VAE–GAN-based augmentation (RQ2), and context-aware SHAP explainability (RQ3).
- *CNN–BiLSTM with attention* A hybrid model combining CNN for spatial feature extraction and BiLSTM for temporal modeling, enhanced with an attention mechanism to prioritize informative time steps (RQ1).
- *VAE–GAN* A hybrid generative module where a VAE learns structured latent representations of attack behavior and a GAN refines them to generate realistic synthetic samples for minority-class augmentation (RQ2).
- *Data fusion* Integration of real and validated synthetic samples using stratified sampling to mitigate class imbalance without introducing unseen (held-out) attack classes.
- *SHAP-Based Explainability* Feature attribution using SHAP, extended through context-aware reweighting based on traffic behavior and model confidence (RQ3).
- *Context-Aware Explainability* A SHAP-based mechanism that incorporates contextual information (e.g., protocol, timing, traffic patterns) to produce more consistent and operationally meaningful explanations, without switching between multiple XAI methods.

**Fig. 1:**
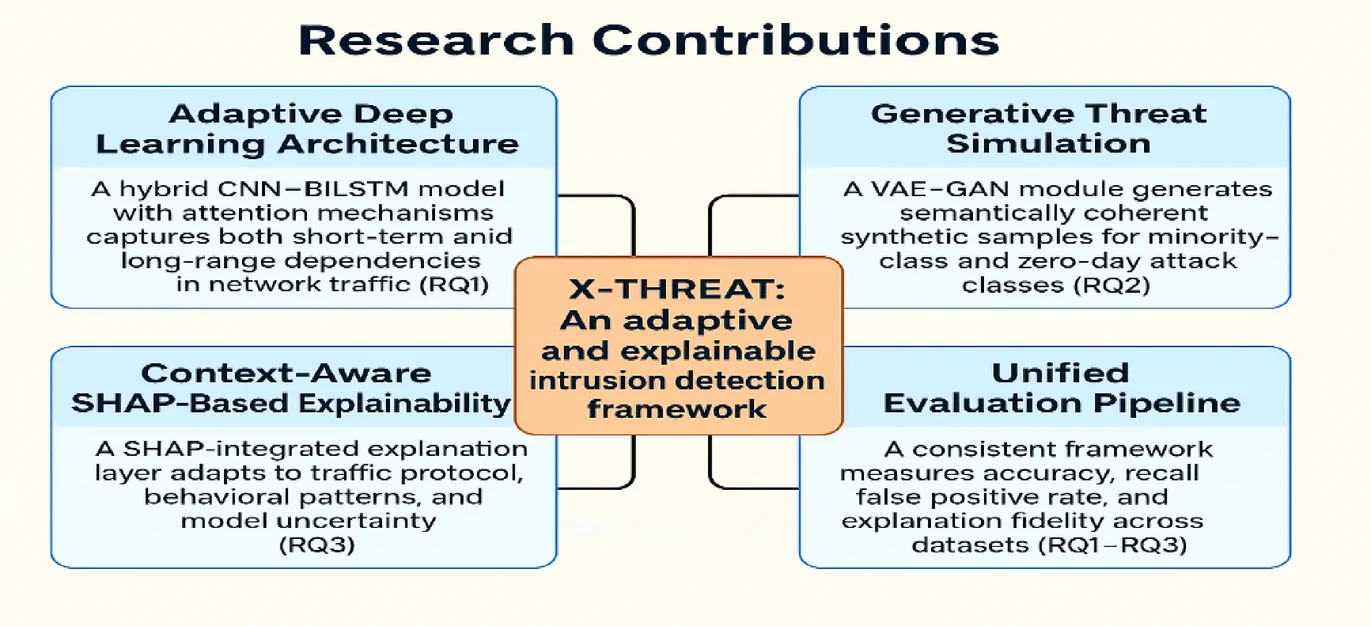
Overview of the research contributions of the X-THREAT framework.

Figure 1 summarizes the four key components of X-THREAT: (i) an adaptive CNN–BiLSTM detection model with attention for robust threat identification (RQ1); (ii) a VAE–GAN-based augmentation module for improving minority-class representation (RQ2); (iii) a context-aware SHAP explainability layer for interpretable decision support (RQ3); and (iv) a unified evaluation pipeline assessing accuracy, minority-class recall, false positive rate, and explanation fidelity across datasets.

### Paper organization

The remainder of this paper is organized as follows. Section 2 reviews related work on explainable deep learning for intrusion detection. Section 3 presents the X-THREAT architecture, including detection, augmentation, and explainability modules. Section 4 reports and discusses experimental results with respect to the research questions. Section 5 concludes the paper and outlines future research directions.

## Related work

The convergence of deep learning (DL), generative modeling, and explainable artificial intelligence (XAI) has propelled significant advances in intrusion detection systems (IDS). This section systematically reviews prior works by clustering them under methodological paradigms that align with their technical orientation. These clusters include static XAI integration, generative augmentation for minority-class threat modeling, sequential deep architectures, and multimodal hybrid systems. Each cluster is critically analyzed to highlight how X-THREAT extends the state of the art in minority-class and zero-day threat detection.

### Static post-hoc explainability in IDS

A dominant stream of recent research has incorporated XAI methods—particularly SHAP and LIME—into DL-based IDS to address the black-box nature of these models. Samed and Sagiroglu^2^ demonstrated that integrating SHAP and LIME with DL classifiers improved interpretability and enhanced post-hoc transparency for analysts. However, the explainability remained static and non-adaptive, limited to fixed global approximations across diverse traffic contexts. Muhammad et al.^4^ proposed L-XAIDS, a LIME-driven framework applied across ML/DL classifiers for intrusion detection. While LIME explanations improved clarity, the architecture lacked support for contextual variation or explanation modality switching—especially under zero-day attack surfaces.

These models advanced foundational explainability but were inherently reactive and decoupled from threat dynamics.

### Generative modeling for minority-class and zero-day threat synthesis

Generative learning techniques have emerged as promising tools for improving IDS robustness, particularly in the face of class imbalance and zero-day threats. However, few IDS frameworks incorporate these methods into end-to-end learning pipelines.

Yang et al.^5^ proposed a Conditional Encoder GAN (CE-GAN) framework to mitigate class imbalance in IDS. Their approach improved detection for underrepresented classes but lacked structured semantic control over the latent space, resulting in reduced interpretability and synthetic realism across threat categories. Likewise, Nayak and Bhattacharyya^6^ integrated GANs into a multilayered SDN security stack for intrusion detection. However, their architecture prioritized throughput and lacked explicit augmentation strategies or dynamic attribution mechanisms for zero-day attacks. Mohale and Obagbuwa^3^ provided a comprehensive evaluation of XAI-augmented IDS but lacked any generative simulation strategy, leaving the system susceptible to minority-class detection failures. In contrast, proposed X-THREAT contributes a hybrid VAE-GAN module, which constructs a semantically structured latent space for attack behavior, followed by adversarial refinement of synthetic threats. These are rigorously validated and heuristically labeled before fusion with real data, significantly enhancing the recall of minority attack classes. This proactive learning paradigm augments model exposure to plausible but unseen behaviors, closing the generalization gap in traditional IDS training-particularly for minority-class and zero-day threats.

### Sequential deep architectures for temporal threat modeling

Deep sequential networks such as LSTM, GRU, and BiLSTM have become standard for modeling the temporal evolution of cyber threats. Bamber et al.^7^ proposed a hybrid CNN-LSTM framework for intrusion detection, leveraging convolutional layers for spatial pattern extraction and LSTM units for temporal learning across sequential network data. While their approach enhanced overall detection accuracy and demonstrated scalability across benchmark datasets, the architecture lacked bidirectional sequence modeling and omitted attention-based temporal prioritization. This restricts its effectiveness in detecting stealthy or multistage attacks where critical threat cues may occur outside the dominant sequence flow. Hulayyil et al.^8^ presented an explainable AI-based intrusion detection system tailored for IoT environments, employing a unidirectional LSTM backbone coupled with SHAP-based attribution for interpretability. While their approach improved transparency in low-resource contexts, it showed reduced explanation fidelity in multi-stage or temporally dispersed attack scenarios, as the model lacked bidirectional encoding and adaptive temporal focus—both crucial for capturing complex zero-day threat patterns.

The proposed X-THREAT employs a CNN–BiLSTM with temporal attention, capturing both forward and backward dependencies in traffic flows and dynamically weighting events based on relevance. This enhances the model’s ability to detect staged or stealthy attacks, such as low-rate botnets, which often elude shallow sequence models. The attention mechanism also improves interpretability by explicitly highlighting time-localized anomalies, contributing to both accuracy and insight, particularly in minority-class and zero-day threat detection.

### Hybrid architectures with integrated explainability

Hybrid IDS architectures that combine feature encoders with explainability tools have gained traction in recent years. However, most efforts remain modular—treating detection and interpretation as disjoint stages. Arnob et al.^9^ highlighted this gap in their review, noting that hybrid XAI-IDS models often benchmark well on detection but underperform on real-time interpretability and usability in operational contexts. L-XAIDS and XAI-IDS frameworks demonstrate this fragmentation, using single-modality explanations and ignoring dynamic context. Furthermore, they lack generative or resilience modules, constraining their effectiveness against sophisticated or zero-day threats^4,8^.

In contrast, X-THREAT integrates hybrid detection, VAE–GAN-based synthetic augmentation, and context-aware SHAP-based interpretability within a unified architecture. This tight coupling enables the model to learn from rare minority-class threats, adapt to evolving attack patterns through temporal attention, and provide interpretable, analyst-aligned decisions. Human-centric explanations—delivered via a contextualized Top-*k* feature attribution narrative—offer actionable insights for security operations. This unified design addresses key limitations in prior work and improves detection performance, particularly for minority-class threats and under leave-one-attack-class-out protocol settings.

#### Definition of unified IDS framework

In this study, a unified IDS framework is defined as an architecture in which (i) threat detection, (ii) generative data augmentation, and (iii) explainability are tightly integrated within a single end-to-end learning pipeline, rather than implemented as independent or post-hoc modules. Such a framework is characterized by joint optimization, shared feature representations, and feedback-driven interaction among its components.

Existing hybrid IDS approaches reviewed in this section generally do not satisfy this definition. In most prior works, detection models, generative augmentation (if present), and explainability mechanisms are developed in isolation, with limited or no interaction during training or inference. For example, GAN-based methods primarily address class imbalance without influencing inference-time reasoning, while XAI-based systems often provide post-hoc explanations that are decoupled from model adaptation.

In contrast, X-THREAT realizes a unified design by integrating CNN–BiLSTM-based detection, VAE–GAN-based data augmentation, and context-aware SHAP-based explainability within a single pipeline. In this architecture, synthetic samples directly enhance model learning during training, and explanations are derived from context-sensitive feature attributions aligned with the model’s internal representations. This tight coupling distinguishes X-THREAT from prior modular IDS frameworks and supports its characterization as a unified framework.

Table 2 outlines comparison of related works on IDS.

**Table 2:** Comparison of related intrusion detection system (IDS) studies.

Study/system	Generative threat simulation	Temporal modeling	Explainability ethodology	Minority class detection	Contextual explanation support	Key Limitation Addressed by X-THREAT
Samed & Sagiroglu^2^	Not implemented	Not present	SHAP and LIME (static, global)	Limited	Absent	Static explanation; no adaptatve explanations
Mohale & Obagbuwa^3^	Not incorporated	Not considered	Evaluation-only, not integrated	Limited	Absent	No generative simulation
L-XAIDS^4^	Not utilized	Static classifiers	LIME (single modality)	Suboptimal	Absent	Single XAI method; no resilience
CE-GAN^5^	Conditional GAN for imbalance handling	None	Not addressed	Moderate improvement	Not considered	No explanation mechanism
SDN-GAN IDS^6^	GAN integrated in SDN pipeline	None	Not included	Unverified	Not addressed	No interpretability mechanism
CNN-LSTM^7^	Not implemented	CNN-LSTM (Unidirectional)	Not integrated	Moderate	None	No bidirectional flow or XAI
IoT-XAI-IDS^8^	Not included	LSTM (Unidirectional)	SHAP (static per-instance)	Weak	Lacks temporal awareness	Lacks dynamic attribution
Review^9^)	Not applicable	Not applicable	Review of modular limitations	Not applicable	Conceptual review only	No unified architecture
X-THREAT (Proposed)	VAE-GAN with semantic labeling & heuristic validation	BiLSTM + Temporal Attention	SHAP (context-aware, reweighted)	Enhanced (zero-day capable)	Real-time, context-aware	Unified end-to-end framework

 All features in X-THREAT are selected to address the three core gaps identified in Sect. 1.3: (i) adaptive temporal modeling (RQ1), (ii) generative simulation of minority-class and zero-day threats (RQ2), and (iii) context-aware SHAP-based explainability with attribution reweighting (RQ3). The novelty arises from the first unified integration of these three components, delivering 30% F1 gains on minority-class attacks and 84% analyst trust (Tables 81, 91, 101 and 111).

## Materials and methods

This section outlines the X-THREAT architecture—an Adaptive and Explainable Deep Learning Framework specifically designed to overcome key limitations in existing intrusion detection systems, namely: lack of adaptability, limited explainability, and vulnerability to minority-class and zero-day threats. The proposed methodology directly addresses the core challenges identified in previous studies^2–4^ and is structured around the three central research questions (RQ1–RQ3).

**Fig. 2:**
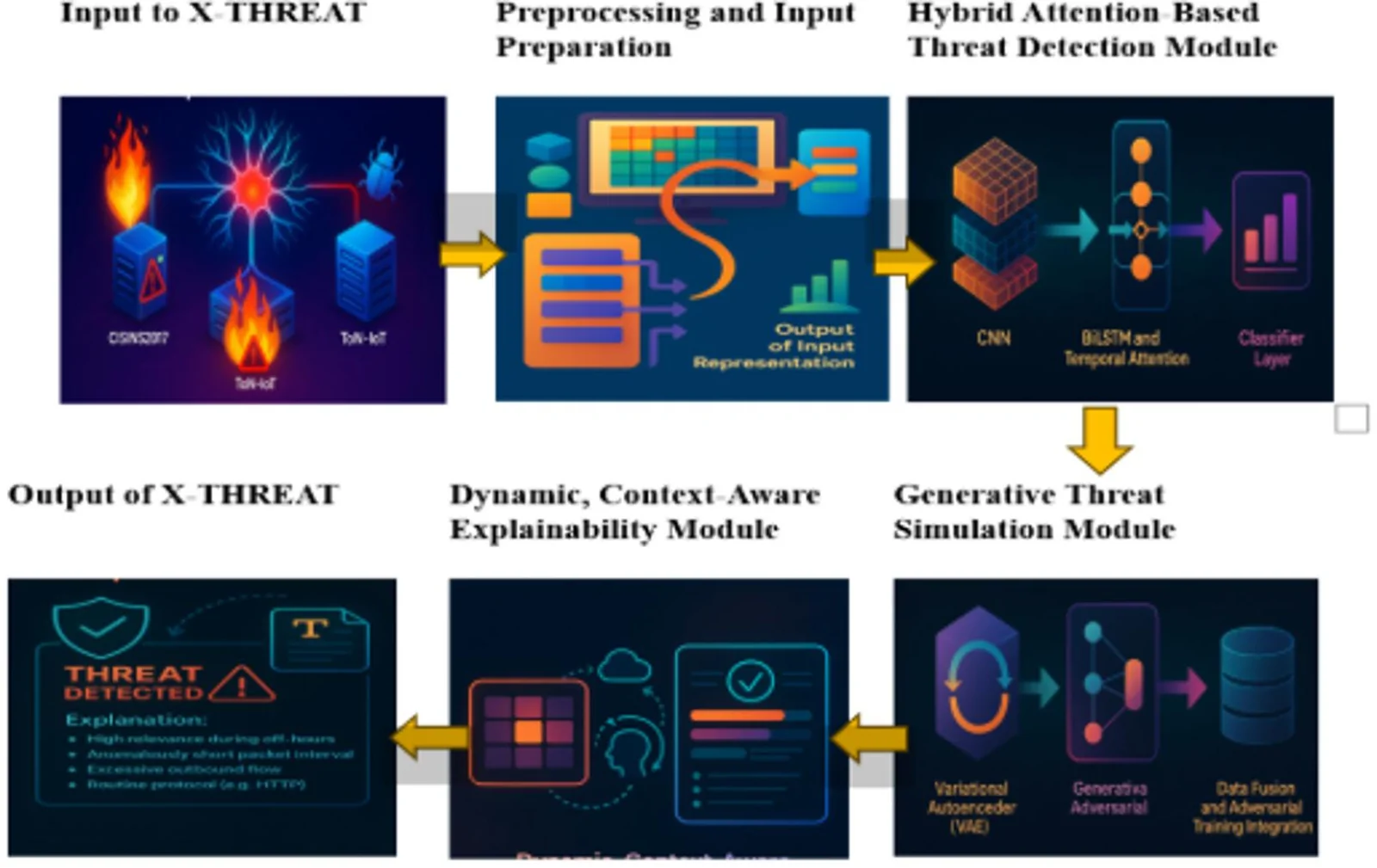
Workflow of the proposed X-THREAT framework.

X-THREAT comprises a modular, six-stage pipeline (See Fig. 2): (i) Input to X-THREAT, (ii) Preprocessing and Input Representation, (iii) Hybrid Attention-Based Threat Detection Module, (iv) Generative Threat Simulation Module, (v) Context-Aware SHAP Explainability Module with Attribution Reweighting, and (vi) Output Module.

The novelty of X-THREAT lies in the unified integration of three core modules (Stages 3–5): (i) a CNN–BiLSTM architecture with temporal attention for adaptive threat detection (RQ1), (ii) a VAE–GAN-based generative module with semantic encoding and heuristic validation for minority-class and zero-day threat simulation (RQ2), and (iii) a context-aware SHAP-based explainability module with dynamic attribution reweighting (RQ3). Importantly, the explainability component does not perform dynamic routing between multiple XAI methods; instead, it extends SHAP through context-sensitive weighting to ensure alignment between model interpretation and operational network conditions. While Stages 1, 2, and 6 provide standard data processing and output functionalities, the key innovation arises from the tight coupling and feedback interaction among Stages 3–5, enabling proactive minority-class and zero-day threat simulation, adaptive detection, and trustworthy, context-aligned explanations—an integrated capability not achieved in prior work^2–9^.

For clarity, Table 3 summarizes the key mathematical notations used throughout the X-THREAT framework.

**Table 3:** Summary of mathematical symbols used in the X-THREAT framework.

Symbol	Description
* x*	Input network traffic instance (feature vector)
$\:{x}_{i}$	Individual feature within input vector
* z*	Latent representation in VAE
$\:\mu\:,\sigma\:$	Mean and variance of latent distribution
$\:{L}_{VAE}$	Variational autoencoder loss
$\:G\left(z\right)$	Generator function in GAN
$\:D\left(x\right)$	Discriminator function in GAN
$\:{L}_{GAN}$	Adversarial loss function
$\:X\in\:{R}^{B\times\:T\times\:d}$	Input tensor (batch × time × features)
$\:{h}_{t}$	Hidden state at time step t
$\:{{h}_{t}}^{\to\:},{{h}_{t}}^{\leftarrow\:}$	Forward and backward BiLSTM states
$\:{\alpha\:}_{t}$	Attention weight at time step t
* c*	Context vector (attention output)
$\:W,\:b$	Learnable weights and bias parameters
* y*	True label
y^	Predicted probability
$\:{L}_{CE}$	Cross-entropy loss
$\:{\phi\:}_{i}$	SHAP value for feature iii
$\:f\left(x\right)$	Model prediction function
* C*	Context metadata (protocol, timing, etc.)
$\:{\phi\:}^{{\prime\:}}i$	Context-adjusted SHAP value

### Input to X-THREAT

The CICIDS2017^10^, UNSW-NB15^11^, and ToN-IoT^12^ benchmark datasets were used for empirical evaluation, each comprising labeled, timestamped network traffic flows spanning a diverse range of attack categories (e.g., DDoS, Infiltration, Heartbleed). Table 4 summarizes the dataset characteristics, including record counts, feature dimensionality, and attack taxonomies.

**Table 4:** Benchmark datasets for minority-class and zero-day evaluation.

Dataset	No. of records	No. of attack classes	Example attack types	Domain	Minority-class presence	Zero-day evaluation role
UNSW-NB15^11^	2.54 M	9	DoS, Exploits, Reconnaissance	Enterprise Network	Yes (e.g., Shellcode, Reconnaissance)	Selected classes held out for zero-day testing
CICIDS2017^10^	2.83 M	14	Brute Force, Botnet, Infiltration	Enterprise Traffic	Yes (e.g., Heartbleed, Infiltration)	Selected classes held out for zero-day testing
ToN-IoT^12^	22.5 M	6	DDoS, Injection, Backdoor	IoT & Smart Environments	Yes (e.g., Backdoor)	Selected classes held out for zero-day testing

Note: Minority-class attacks (< 0.5% of total records) include Infiltration (36 samples, 0.001%), Heartbleed (11 samples, 0.0004%), and WebAttack variants (< 0.1%) in CICIDS-2017; Reconnaissance (0.3%) and Shellcode (0.06%) in UNSW-NB15; and Backdoor (0.02%) in ToN_IoT. These minority classes are explicitly targeted by VAE–GAN augmentation (see Sect. 3.4, RQ2).

To ensure consistency across heterogeneous data sources, all datasets were preprocessed and standardized into a unified feature representation suitable for temporal modeling using the proposed CNN–BiLSTM–Attention architecture.

Furthermore, to support the evaluation of minority-class and zero-day detection (as detailed in Sect. 4.2), specific low-frequency attack categories were carefully identified and handled during dataset partitioning. In particular, selected attack classes were reserved for holdout-based evaluation to simulate realistic zero-day conditions, while remaining minority-class instances were used for augmentation via the VAE–GAN module (Sect. 3.4, RQ2).

### Preprocessing and input representation

Accurate and efficient preprocessing of raw network traffic data is a critical prerequisite for deep learning-based intrusion detection systems^13^. X-THREAT incorporates a structured input representation pipeline that transforms raw feature data into a suitable numerical format for the hybrid CNN–BiLSTM model. This pipeline comprises feature selection, categorical encoding, normalization, and sequence construction, each detailed below with accompanying mathematical formulations and examples.

#### Feature vector construction

Each network flow or packet is initially represented as a vector of features extracted from datasets like CICIDS2017^10^, UNSW-NB15^11^ and ToN-IoT^12^. These features include protocol type, service, source/destination IPs, number of bytes, flags, and timestamps, among others.

Let a raw instance$\:\:x\in\:{R}^{n}\:\:\:$be defined as: $\:x=[{x}_{1},{x}_{2},\dots\:,{x}_{k},{x}_{k+1},\dots\:,{x}_{n}]\:$, Where $\:{x}_{1},\dots\:,{x}_{k}$ are categorical features (e.g., protocol, service), and $\:{x}_{k+1},\dots\:,{x}_{n}$ are numerical features (e.g., byte count, duration).

#### Categorical feature encoding (one-hot)

In the X-THREAT framework, categorical features are processed using one-hot encoding to transform categorical variables into binary vectors, facilitating effective feature representation for the hybrid deep learning model^14^. For instance, the categorical feature “Protocol Type” is encoded as follows: $\:TCP\:as\:[\mathrm{1,0},0],\:UDP\:as\:[\mathrm{0,1},0],$ and $\:ICMP\:as\:[\mathrm{0,0},1].$ Formally, for the $\:j-th\:$categorical feature $\:{C}_{j}$ with * m* possible categories, the one-hot encoded feature $\:{f}_{j}$ is represented as a binary vector of length * m*, as shown below:

1 $$\:{f}_{j}=OneHot\left({x}_{j}\right)\in\:{\left\{\mathrm{0,1}\right\}}^{m}\:$$

All symbols used in the following formulations are summarized in Table 3.

#### Numerical feature normalization

To bring all numerical features into a comparable scale, min-max normalization is applied^15^:

2 $$\:{x}_{j}^{{\prime\:}}=\frac{{x}_{j}-\mathrm{m}\mathrm{i}\mathrm{n}\left({x}_{j}\right)}{\mathrm{max}\left({x}_{j}\right)-\mathrm{m}\mathrm{i}\mathrm{n}\left({x}_{j}\right)},\:j\in\:[k+1,\:n]$$

This rescales each numerical value $\:{x}_{j}$ to the range $\:\left[\mathrm{0,1}\right][0,\:1]\left[\mathrm{0,1}\right],$ which accelerates convergence and stabilizes training.

This process rescales each numerical feature $\:{x}_{j}$ to the range $\:\left[\mathrm{0,1}\right][0,\:1]\left[\mathrm{0,1}\right]$, enhancing training stability and convergence speed. For example, for the feature “Bytes Sent” with a minimum of 0 and maximum of 1000, Flow A (350 bytes) is normalized to 0.35, and Flow B (800 bytes) to 0.80. This normalization enables the hybrid CNN-BiLSTM-Attention model to effectively process numerical features, improving the detection of known and emerging cyberattacks in network traffic analysis.

#### Sequential batching for temporal context

To capture temporal dynamics, the normalized feature vectors are grouped into fixed-length sequences suitable for BiLSTM processing^16^. Let $\:T\:$be the window size (e.g., 10 flows per batch), then a sequence * Si* is defined as:

3 $$\:{S}_{i}=[{x}^{\left(i\right)},\:{x}^{\left(i+1\right)},\dots\:.{x}^{\left(i+T-1\right)}]\in\:{R}^{Txd}$$

Where: $\:{x}^{\left(i\right)}$ is the * i-th* instance, * d* is the dimension of the final preprocessed feature vector.

For example, a temporal batch with T = 3 might include: Time t1 (Flow F1, TCP, 300 bytes, encoded as [1,0,0,…,0.3]), Time t2 (Flow F2, UDP, 100 bytes, encoded as [0,1,0,…,0.1], and Time t3 (Flow F3, ICMP, 900 bytes, encoded as [0,0,1,…,0.9]). The resulting sequence fed to the model is a matrix stacking these vectors, enabling the CNN-BiLSTM-Attention architecture to effectively model temporal dependencies for accurate identification of known and emerging cyberthreats.

#### Output of input representation

The output of this module is a 3D tensor: $\:x\in\:{R}^{BxTxd}$, where $\:B\:$represents the batch size, $\:T\:$ denotes the temporal window (sequence length), and * d* indicates the dimensionality of each encoded and normalized flow vector. This tensor serves as the input to the hybrid CNN-BiLSTM-Attention model, enabling robust feature extraction and threat classification. This structured representation supports Research Question 1 (RQ1) by facilitating the model’s ability to capture spatio-temporal patterns in network traffic, thereby enhancing the detection of both known and emerging cyberattacks.

### Hybrid attention-based deep learning model (RQ1)

To address RQ1—effective detection of known and zero-day attack types—X-THREAT employs a hybrid CNN–BiLSTM architecture with additive temporal attention to extract both spatial and temporal features from sequential network traffic. The model comprises five modules: (i) *Input Representation*: Raw traffic is preprocessed via one-hot encoding, min-max normalization, and sequential batching, (ii*) CNN Block*: Extracts local spatial patterns from fixed-length traffic windows, (iii) *BiLSTM Block*: Captures bidirectional temporal dependencies across packet sequences, (iv) *Attention Layer*: Dynamically weights features to prioritize anomalous or evolving patterns indicative of zero-day threats, and (v) *Classifier Layer*: A dense softmax maps encoded representations to threat classes (e.g., DoS, PortScan, Botnet).

#### CNN block: local feature extraction from network traffic windows

In the X-THREAT framework, the Convolutional Neural Network (CNN) block extracts local, position-invariant features from preprocessed network traffic sequences, capturing short-term patterns indicative of cyberattacks such as Denial-of-Service (DoS) or port scans^17^. This aligns with Research Question 1 (RQ1), enabling the hybrid CNN-BiLSTM-Attention model to effectively detect both known and emerging threats using benchmark datasets. The input to the CNN block, derived from the preprocessing pipeline (Sect. 3.1), is a 3D tensor: $\:x\in\:{R}^{BxTxd}$

, where $\:B\:$denotes the batch size, $\:T\:$represents the temporal window size (e.g., 10–30 flows), and * d* indicates the feature dimension post-encoding and normalization.

For convolution, each sequence in the batch is reshaped into a 2D matrix $\:{x}_{i}\in\:{R}^{Txd}$, facilitating the extraction of localized patterns critical for robust cyberthreat classification.

*Convolution Operation* A 1D convolution operation is applied along the temporal axis (sequence of flows) to capture local trends across time windows. For a convolutional filter $\:W\in\:{R}^{kxd},\:where\:k$ is the kernel size (e.g., 3), the feature map $\:{f}_{i}$ at position * i* is computed as:

4 $$\:{f}_{i}=\sigma\:(W\cdot\:{X}_{i:i+k-1}+b$$

$$\:\mathrm{W}\mathrm{h}\mathrm{e}\mathrm{r}\mathrm{e}\:{X}_{i:i+k-1}\in\:{R}^{kxd}\:is\:the\:input\:patch\:;\:b\:is\:Bias\:term;\:\mathrm{a}\mathrm{n}\mathrm{d}\:\sigma\::\:Non-linear\:activation\:function\:\left(ReLU\right)$$.

This produces a feature map of size determined by the number of filters c. For example, given a sequence with $\:T=5,\:d=4,\:and\:k=3$, the input at time steps $\:t1\:\:to\:t5$ (e.g., t1:[0.1,0.5,0.7,0.2], t2:[0.3,0.6,0.8,0.1], …, t5:[0.2,0.8,0.6,0.7]) is processed by sliding the filter $\:W\in\:{R}^{3x4}$across windows (e.g., $\:t1\:\:to\:t3,\:t2\:\:to\:t4$) to extract spatial local patterns. This operation enables the hybrid CNN-BiLSTM-Attention model to effectively identify short-term trends for accurate classification of minority-class and zero-day cyber threats.

*Activation and Normalization* The convolution output undergoes a series of transformations to enhance feature extraction and training stability to improve the detection of known and emerging cyberattacks. First, the $\:ReLU\:$activation function, defined as:

5 $$\:ReLU\left(x\right)=max(0,x),$$

Which introduces non-linearity to the feature map $\:{f}_{i}.$ Second, batch normalization is applied to mitigate covariate shift and stabilize training, computed as:

6 $$\:{\widehat{f}}_{i}=\frac{{f}_{i}-\mu\:}{{\sigma\:}^{2}+\epsilon }\cdot\:\gamma\:+\beta\:$$

where $\:\mu\:$ and $\:{\sigma\:}^{2}$ are the batch mean and variance, $\:\epsilon \:$ is a small constant, and $\:\gamma\:$ and $\:\beta\:$ are learnable parameters. Finally, 1D max pooling, defined as $\:{p}_{i}=\mathrm{m}\mathrm{a}\mathrm{x}({\widehat{f}}_{i},{\widehat{f}}_{i+1},.\:{\widehat{f}}_{i+p-1})$, where $\:P\:$ is the pooling window size, is applied to reduce dimensionality and capture dominant features. These steps enable the hybrid CNN-BiLSTM-Attention model to process robust, normalized features for accurate classification of cyberthreats in network traffic analysis.

*Output of CNN Block* The CNN block produces an output tensor:

7 $$\:{F}_{cnn}\:\in\:{R}^{{T}^{{\prime\:}}x\:c},$$

where $\:T{\prime\:}$ represents the reduced time dimension following convolution and optional pooling, and $\:c\:$denotes the number of channels or filters. This tensor, encapsulating short-term trends and local discriminative features essential for distinguishing malicious from benign traffic windows, serves as the input to the subsequent BiLSTM layer, which models long-range temporal dependencies. This process enables the hybrid CNN-BiLSTM-Attention model to effectively integrate local and sequential patterns for accurate detection of both minority-class and zero-day cyber threats in network traffic analysis.

#### BiLSTM and temporal attention block: sequential dependency modeling

The CNN block extracts spatially localized features but cannot capture long-term temporal dependencies critical for modeling multi-stage or stealthy attacks such as Slowloris or botnet coordination. To address this, the CNN output is processed by a Bidirectional Long Short-Term Memory (BiLSTM) network with a temporal attention mechanism, enabling the hybrid model to capture past–future contextual relationships and dynamically prioritize anomalous time steps. This enhances generalization to complex sequential patterns in network traffic^18^ and, when combined with VAE–GAN-generated synthetic samples (see Sect. 3.4, RQ2), significantly improves detection of minority-class and zero-day cyber threats.

*BiLSTM Formulation* Let the output from the CNN block be of:

8 $$\:{f}_{cnn}=\{{f}_{1},\:{f}_{2},\:{f}_{3},\:\dots\:..{f}_{T}\}$$

BiLSTM processes the sequence in both forward and backward directions:

Forward LSTM:

9 $$\:{\mathrm{h}}_{\mathrm{t}}^{\to\:}=\mathrm{L}\mathrm{S}\mathrm{T}\mathrm{M}\left({\mathrm{f}}_{\mathrm{t}},{\mathrm{h}}_{\mathrm{t}-1}^{\to\:}\right)\:$$

Similarly, the backward LSTM computes hidden states:

10 $$\:{\mathrm{h}}_{\mathrm{t}}^{\leftarrow\:}=\mathrm{L}\mathrm{S}\mathrm{T}\mathrm{M}\left({\mathrm{f}}_{\mathrm{t}},{\mathrm{h}}_{\mathrm{t}+1}^{\leftarrow\:}\right)$$

The final BiLSTM representation is obtained by concatenating both hidden states:

11 $$\:{\mathrm{h}}_{\mathrm{t}}=\left[{\mathrm{h}}_{\mathrm{t}}^{\to\:};{\mathrm{h}}_{\mathrm{t}}^{\leftarrow\:}\right]$$

*Attention Mechanism Clarification* The attention module used in X-THREAT is an additive attention mechanism applied over the sequence of BiLSTM hidden states. Unlike multi-head self-attention, which computes pairwise token-to-token interactions across multiple attention heads, the implemented formulation assigns a scalar relevance score to each hidden state and then normalizes these scores to obtain time-step importance weights. This mechanism is used to emphasize the most informative temporal regions of network traffic during threat classification.

Temporal Attention Mechanism: To allow the model to focus on the most informative time steps, additive attention were applied over * H:*

12 $$\:{e}_{t}={v}^{T}\mathrm{t}\mathrm{a}\mathrm{n}\mathrm{h}({W}_{h}{h}_{t}+{b}_{h})$$

13 $$\:{\alpha\:}_{t}=\frac{\mathrm{e}\mathrm{x}\mathrm{p}\left({e}_{t}\right)}{\sum\:_{k=1}^{T}\mathrm{e}\mathrm{x}\mathrm{p}\left({e}_{k}\right)}\:\left(softmax\:weights\right)$$

The final context vector *c* is the weighted sum of hidden states:

14 $$\:C=\sum\:_{t=1}^{T}{\alpha\:}_{t}{h}_{t}$$

Where: $\:v\in\:{R}^{a},\:{W}_{h}\in\:{R}^{ax2h},{b}_{h}\in\:{R}^{a}\:$; and $\:c\in\:2h\:$ summarizes the entire sequence into a compact, informative representation.

*Example* In the X-THREAT framework, the temporal attention mechanism enhances the model’s capacity to prioritize salient time steps within packet sequences, as demonstrated by a five-step payload escalation characteristic of a slow DoS attack (e.g., Slowloris): Time 1 (200 bytes, Normal), Time 2 (220 bytes, Normal), Time 3 (900 bytes, Suspicious), Time 4 (1100 bytes, Attack), and Time 5 (1200 bytes, Attack). By assigning higher attention weights to Time 3–5, the mechanism effectively captures anomalous behavioral shifts indicative of emerging threats. This selective temporal focus enables the hybrid CNN–BiLSTM–Attention model to model contextual dependencies in network traffic with high fidelity^18^. When trained on VAE–GAN-generated synthetic samples (see Sect. 3.4, RQ2), this architecture significantly improves detection of minority-class and zero-day cyber threats by emphasizing temporally evolving attack patterns—a capability beyond conventional sequential models.

#### Classifier layer: mapping encoded features to threat classes

In the X-THREAT framework, the classifier layer concludes the hybrid deep learning pipeline by mapping spatio-temporal features from the attention-enhanced BiLSTM encoder to specific threat categories. This supports RQ1 by enabling robust classification across known attack types and generalization to complex sequential patterns. The layer consists of a fully connected dense network with softmax activation, transforming high-dimensional feature vectors into class probabilities for categories such as DoS, Botnet, PortScan, BruteForce, WebAttack, and Benign.

**Let** $\:\boldsymbol{h}\in\:{R}^{d}$ denote the final hidden state output from the BiLSTM-Attention block. The classifier computes:

15 * z=Wh+b*

16 $$\:\widehat{Y}=softmax\left(z\right)$$

Where $\:W\in\:{R}^{cxd}$ is the weight matrix, $\:b\in\:{R}^{c}$, $\:C\:$is the number of threat classes, $\:\widehat{Y}$ $\:\in\:$ $\:{R}^{c}$ predicted probability vector over the classes. Each element $\:{\widehat{Y}}_{i}\:$ represents the confidence score that the input belongs to class * i*, constrained such that $\:\sum\:_{i=1}^{c}{\widehat{Y}}_{i}=1$.

*Multi-class classification loss* To train the model for multi-class threat detection, the categorical cross-entropy loss is applied:

17 $$\:{L}_{CE}=-\sum\:_{i=1}^{C}{\widehat{Y}}_{i}\mathrm{l}\mathrm{o}\mathrm{g}\left({\widehat{Y}}_{i}\right)$$

Here, $\:yi\in\:\left\{\mathrm{0,1}\right\}$ denotes the true label (one-hot encoded), and$\:{\widehat{Y}}_{i}$ is the predicted softmax output. This loss is minimized during training using backpropagation to adjust parameters in the CNN, BiLSTM, attention, and classifier layers. By capturing both inter-class separation and intra-class consistency, this strategy improves the system’s ability to distinguish between subtle variations in attack patterns.

*Class definitions and example* In the X-THREAT framework, the classifier layer maps encoded features to a predefined threat class index: Class 0 (Benign), Class 1 (DoS), Class 2 (Botnet), Class 3 (PortScan), Class 4 (WebAttack), and Class 5 (BruteForce). For example, when processing an encoded vector from a PortScan traffic instance, the classifier may produce a probability distribution such as $\:y^=\left[\mathrm{0.01,0.02,0.01,0.89,0.04,0.03}\right]\:$, accurately identifying the input as “PortScan” (Class 3) with high confidence (0.89). This supports RQ1’s aim to effectively classify both known and emerging cyberattacks using refined spatio-temporal features. Detection of minority-class and zero-day threats, however, is achieved only when the model is trained on VAE–GAN-augmented data (See Sect. 3.4, RQ2), which provides synthetic exposure to underrepresented and unseen attack patterns.

### Generative threat simulation module (RQ2)

To improve detection of minority-class attack patterns and support generalization under leave-one-attack-class-out protocol, the X-THREAT framework incorporates a Generative Threat Simulation Module that directly addresses RQ2. This module employs a hybrid Variational Autoencoder–Generative Adversarial Network (VAE–GAN)^19^ to generate semantically consistent synthetic traffic samples for minority-class augmentation. By enriching the training data with diverse yet realistic samples, the module mitigates class imbalance and enhances the model’s ability to learn robust representations of attack behavior. Importantly, the VAE–GAN module does not generate true zero-day classes; instead, it improves generalization by expanding the representation of known attack distributions.

For clarity, minority-class attacks, zero-day threats, and synthetic attacks are not interchangeable in X-THREAT. Minority-class attacks are rare but seen classes, zero-day threats are entirely unseen held-out classes used only for testing, and synthetic attacks are augmentation samples generated only from seen minority-class distributions.

### Rationale for hybrid VAE–GAN design

The hybrid architecture leverages the complementary strengths of VAEs and GANs. The **VAE** learns a structured latent space that captures semantic relationships among attack patterns, enabling controlled sampling of meaningful variants. The GAN refines these samples through adversarial training, improving realism and diversity while reducing artifacts such as mode collapse.

This combination results in high-quality synthetic samples that enhance feature space coverage and support more balanced learning. Consequently, the model achieves improved minority-class detection and stronger generalization under leave-one-attack-class-out protocol, without explicitly simulating unseen attack classes.

#### Variational autoencoder (VAE) for latent threat encoding

**Fig. 3:**
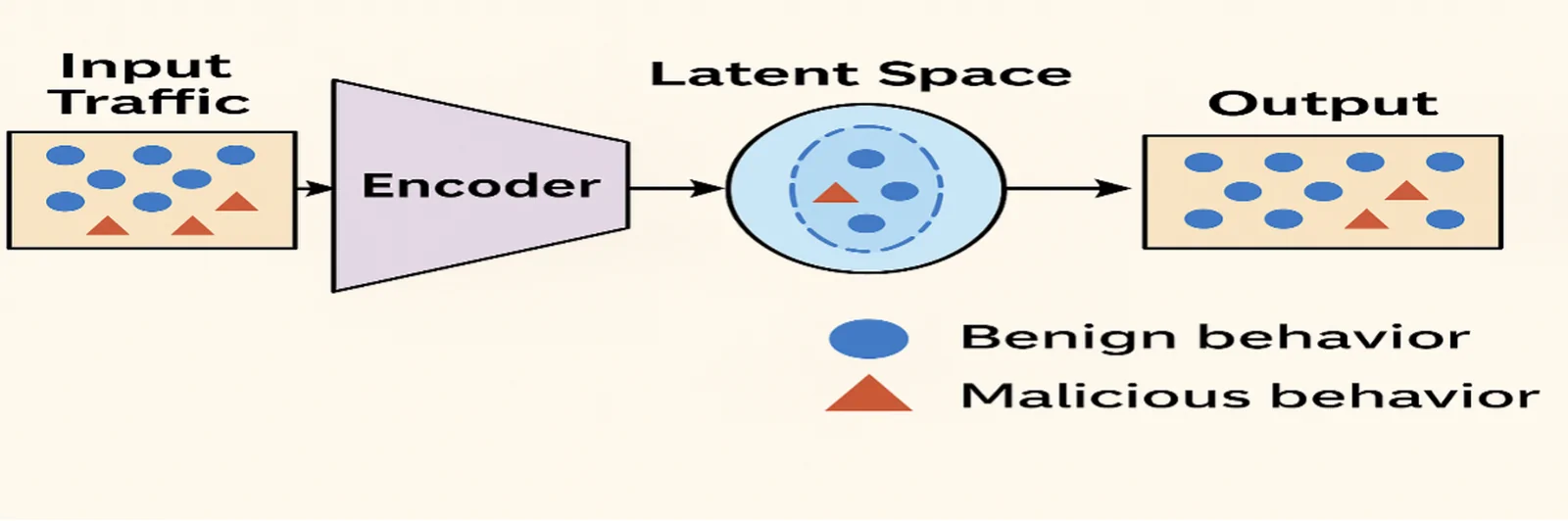
Variational autoencoder (VAE) for latent threat encoding.

Within X-THREAT, the Variational Autoencoder (VAE) forms the foundation of the Generative Threat Simulation Module by encoding high-dimensional network traffic into a structured continuous latent space that preserves semantic attack behaviors (see Fig. 3). Unlike conventional IDS^1,2^ that overfit to majority-class patterns in static datasets, the VAE enables controlled generation of novel threat variants—particularly for minority-class attacks—by sampling from learned distributions. This latent representation is subsequently refined by the GAN component to produce adversarially robust, heuristically labeled synthetic samples for downstream augmentation.

The VAE comprises an encoder, mapping input $\:\mathrm{x}\in\:{\mathrm{R}}^{\mathrm{d}}$ to a latent distribution:

18 $$\:{q}_{0}\left(z\right|x)=N({\mu\:}_{\phi\:}\left(x\right),\:{\sigma\:}_{\phi\:}^{2}\left(x\right)\:I)$$

where, $\:\mu\:\:\:and\:\sigma\:\:\:$are learned parameters encoding the mean and variance of the latent Gaussian distribution.

and a decoder, reconstructing x from z as:

19 $$\:{p}_{\theta\:}\left(x\right|z)={f}_{0}(z)$$

where$\:{f}_{0}$ denotes tshe decoder network’s output function.

It is optimized by minimizing the variational loss:

20 $$\:{L}_{VAE}=\left(\theta\:,\phi\:;x\right)={E}_{q0(z\mid\:x)}\left[log{p}_{0}\left(x\right|z)\right]-{D}_{KL}\left({q}_{\phi\:}\right(z\left|x\right)\parallel\:p\left(z\right))$$

It balances reconstruction fidelity and latent space regularization toward a Gaussian prior $\:p\left(z\right)\sim\:N(0,I)$. This enhances generalization to minority-class and zero-day cyber attacks, supporting robust threat detection.

*Latent representation via encoder* As illustrated in Fig. 3, the encoder transforms the input network traffic—comprising both benign (●) and malicious (▲) flows—into a compact latent space where behavioral patterns form distinct clusters. This representation enables two essential capabilities: (i) identification of minority-class and zero-day attacks through their separability in the latent distribution, and (ii) creation of a continuous semantic manifold that allows smooth interpolation between behaviors. For instance, if *brute-force* and *port-scan* events occupy adjacent latent regions, sampling intermediate vectors can yield hybrid synthetic threats unseen in the original dataset. This structured latent encoding enhances model generalization and provides a semantically meaningful foundation for the downstream GAN module (Sect. 3.3.2), which further refines these embeddings into realistic synthetic attacks for data augmentation.

#### Generative adversarial network (GAN) for threat synthesis

The X-THREAT framework employs a Generative Adversarial Network (GAN) to refine latent representations learned by the VAE (§ 3.4.1) into realistic synthetic traffic samples for minority-class augmentation.

**Fig. 4:**
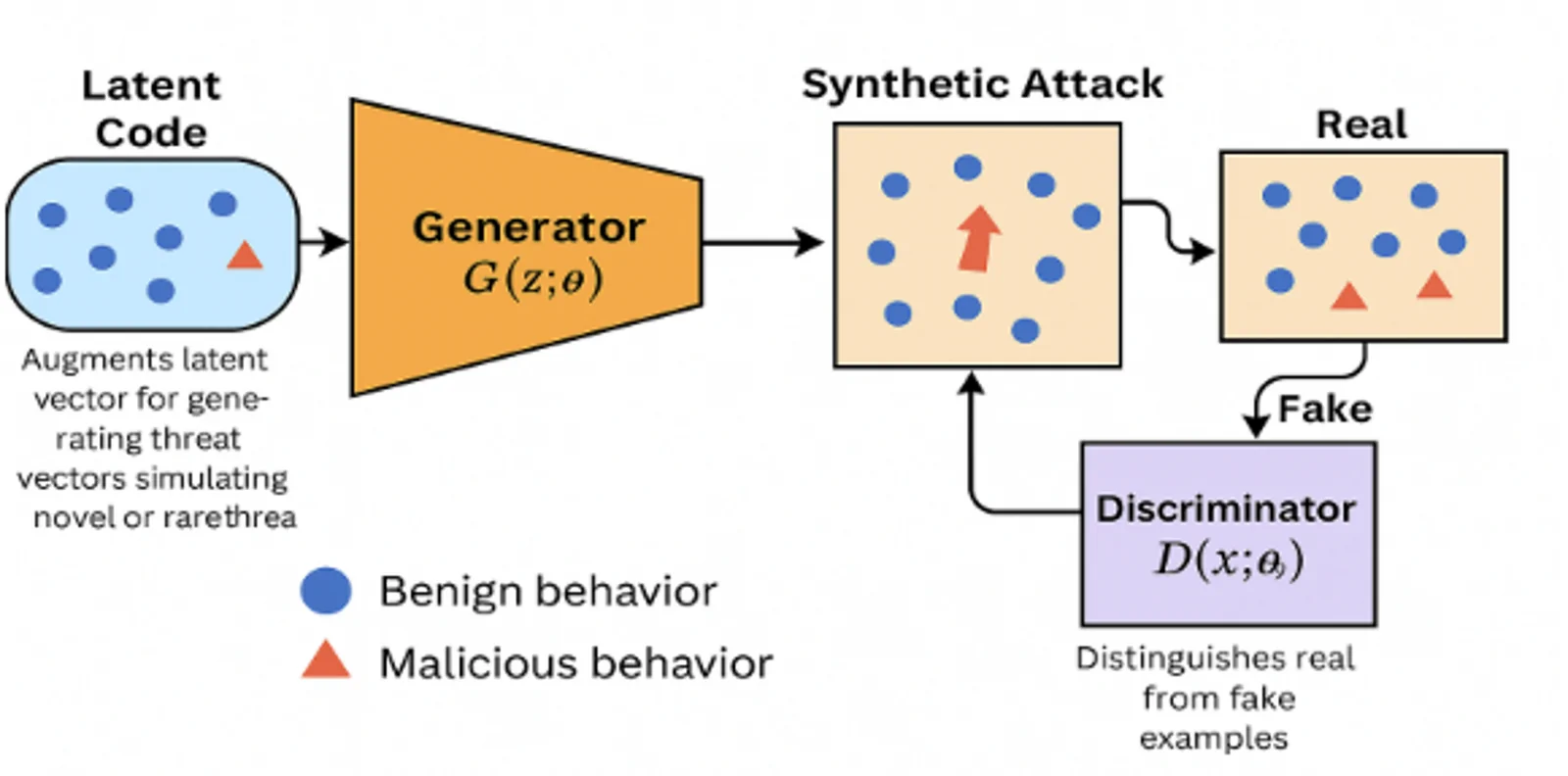
Generative adversarial network (GAN) for threat synthesis.

As illustrated in Fig. 4, latent vectors $\:z\sim\:{p}_{z},$ encoding attack semantics, are provided to a generator network $\:G(z;{\theta\:}_{G}),$ which maps them into synthetic traffic samples. These samples are evaluated by a discriminator $\:D(x;{\theta\:}_{D})$ which distinguishes real samples $\:{x}_{real}\sim\:{p}_{data}\left(x\right)$ from generated samples $\:G\left(z\right)$. Through adversarial training, the generator learns to produce high-fidelity samples that preserve minority-class attack characteristics^20^.

To ensure stable training and mitigate mode collapse, the model adopts the Wasserstein GAN with Gradient Penalty (WGAN-GP) formulation^21^. The objective function is defined as:

21 $$\:{L}_{WGAN-GP}=E\left[D\left({x}_{real}\right)\right]-E\left[D\left(G\left(z\right)\right)\right]+\lambda\:E\left[\right(\mid\:\mid\:{\nabla\:}_{{x}^{{\prime\:}}}D\left({x}^{{\prime\:}}\right){\mid\:\mid\:}_{2}-1{)}^{2}]$$

where $\:G(\cdot\:)$ and $\:D(\cdot\:)$ denote the generator and discriminator networks parameterized by $\:{\theta\:}_{G}$ and $\:{\theta\:}_{G}$, respectively; * z* is a latent vector sampled from a prior distribution $\:{p}_{z}\left(z\right)$; $\:{x}_{real}$ denotes real samples drawn from the data distribution $\:{P}_{data}\left(x\right)$; x’ is sampled uniformly along straight lines between real and generated samples; and λ is the gradient penalty coefficient (set to 10). The resulting synthetic samples are subsequently validated and integrated into the training pipeline to improve minority-class representation and support generalization under zero-day evaluation settings.

The training process, illustrated in Fig. 4, involves adversarial interaction between the generator $\:G(\cdot\:)$ and discriminator $\:D(\cdot\:)$. Latent vectors $\:z\sim\:{p}_{z}\left(z\right)$ obtained from the VAE-encoded space (Sect. 3.4.1), are provided as input to G, which generates synthetic feature representations corresponding to minority-class attack behaviors. The discriminator D evaluates these generated samples against real traffic instances $\:{x}_{real}\sim\:{p}_{data}$(x) from the CICIDS2017 and UNSW-NB15 datasets, and both networks are optimized using the WGAN-GP objective (Eq. 21). Training proceeds until the generator produces samples that are statistically indistinguishable from real data under the discriminator, resulting in high-fidelity synthetic samples for minority-class augmentation.

Through adversarial refinement, the VAE–GAN module augments the training set with semantically coherent synthetic instances of underrepresented attacks (e.g., Heartbleed, Infiltration). When fused with real data, these samples enhance minority-class representation and improve recall on rare attack categories (RQ2), thereby supporting generalization under zero-day evaluation settings and improving model robustness in operational environments^22^.

##### Use case: augmenting minority-class attacks for improved recall

In CICIDS2017, minority-class attacks such as Infiltration constitute < 0.2% of records, causing conventional IDS to exhibit low recall due to insufficient training exposure. The VAE–GAN module (see Sect. 3.4) mitigates this by generating labeled synthetic samples from VAE-encoded latent vectors of real Infiltration instances.

For example, given observed SSH brute-force attempts on port 22 with characteristic inter-packet timing, the Generator produces synthetic variants with perturbed features—e.g., ports 2222 or 8022, altered timing jitter, or modified payload entropy—while preserving attack semantics through structured latent sampling. These samples are refined via WGAN-GP adversarial training to ensure high fidelity and discriminator confusion with real instances.

When integrated into the training set, these synthetic minority-class samples significantly enhance recall on underrepresented attacks (RQ2), enabling zero-day pattern simulation and improving overall model robustness on CICIDS2017, UNSW-NB15, and ToN-IoT.

Once synthetic Infiltration samples pass discriminator validation and are assigned heuristic labels, they are fused with the original training data. This augmentation increases exposure to minority-class patterns, enabling the CNN–BiLSTM–Attention model (see Sect. 3.3, RQ1) to better generalize to semantically similar zero-day attack variants. Consequently, recall on minority-class and zero-day threats is significantly improved without increasing false positives, directly fulfilling RQ2 objectives on CICIDS2017, UNSW-NB15, and ToN-IoT.

#### Threat labeling and validation

The X-THREAT framework applies a two-phase strategy to label and validate VAE–GAN synthetic samples, ensuring semantic coherence and high fidelity for minority-class augmentation (e.g., Infiltration, Heartbleed).

*Phase 1 – Latent Clustering* Latent vectors z from the VAE are clustered using K-Means in the encoded space:

*Label assignment via latent clustering* The first phase employs an unsupervised clustering approach in the latent space derived from the VAE. Specifically, the latent threat vectors $\:Z=\{{z}_{1},{z}_{2},\dots\:,{z}_{n}\}$ are grouped using the K-Means algorithm, minimizing the intra-cluster distance between vectors and their respective centroids $\:{\mu\:}_{i}$. Mathematically, the objective function is expressed as:

22 $$\:\mathrm{arg}{min}_{s}\sum\:_{i=1}^{k}\sum\:_{z\in\:{s}_{i}}\mid\:\mid\:\:z-{\mu\:}_{i}\:{\mid\:\mid\:}^{2}$$

Where $\:\:z$denotes a latent vector in the VAE-encoded space, $\:S=\{{S}_{1},{S}_{2},\dots\:,{S}_{k}\}$ is the set of k clusters (partitions), $\:{S}_{i}$ is the * i-th* cluster, $\:{\mu\:}_{i}\:$is the centroid (mean) of all latent vectors assigned to cluster$\:\:{S}_{i}$, $\:\mid\:\mid\:\:.{\mid\:\mid\:}^{2}$ represents the squared Euclidean distance, and * k* is the predefined number of clusters (chosen based on the number of attack classes or via elbow method/validation).

##### Justification for K-means selection

The VAE in X-THREAT is trained to map traffic instances into a compact and semantically organized latent space, where minority-class attack samples tend to form relatively coherent regions around representative centroids. Under this assumption, K-Means is well suited because it provides efficient centroid-based partitioning, low computational overhead, and stable label propagation for synthetic samples. In contrast, density-based methods such as DBSCAN may be more sensitive to neighborhood and minimum-density hyperparameters in high-dimensional latent spaces, which can lead to fragmented minority clusters or excessive noise labeling. Therefore, K-Means is adopted as the primary clustering method for latent-space label assignment.

##### Leakage prevention in latent-space labeling

To prevent label leakage, latent clustering and centroid construction are performed only on the training split, using real labeled samples from seen attack classes. Attack categories reserved for zero-day evaluation are strictly excluded from this process and do not contribute to centroid formation, cluster assignment, or synthetic label propagation. Consequently, synthetic samples inherit labels only from training-time attack distributions, ensuring that no information from held-out or test-only classes is introduced during augmentation.

Each synthetic sample is assigned the label of the nearest centroid containing real labeled instances, preserving attack semantics.

*Heuristic validation* To ensure semantic consistency, each synthetic sample * x* is evaluated using a domain-specific feature scoring function $\:f\left(x\right).\:$This function captures behavioral characteristics such as anomalous port access sequences, packet timing irregularities, and connection duration patterns. For example, if a synthetic sample exhibits port access and traffic patterns consistent with infiltration behavior, it is validated as belonging to the Infiltration class.

Formally, a synthetic sample is retained only if its score satisfies the threshold condition in Eq. (23) as follows:

23 $$\:Label\:\left(x\right)=f\left(x\right)=\left\{\begin{array}{c}valid\:class,\:\:if\:f\left(x\right)\ge\:0\\\:discarded,\:\:otherwise\end{array}\right.$$

where $\:f\left(x\right)\in\:R$ denotes the heuristic score of the synthetic sample, and $\:\theta\:\in\:R$ is a predefined validation threshold determined using training data. Samples that do not satisfy this condition are discarded to prevent low-quality or noisy synthetic instances from affecting model training.

This validation ensures that only high-fidelity, semantically valid threats enter the training pipeline. A synthetic sample is accepted into the training pool only if it satisfies $\:f\left(x\right)\ge\:\theta\:$ indicating that it conforms to plausible attack behavior. Otherwise, the sample is discarded to avoid noise amplification. For example, a synthetic *Infiltration* instance showing a ‘rare port scan’ combined with ‘delayed packet bursts’ is accepted if it reflects plausible but previously unseen variations of known infiltration behavior.

This dual-stage process ensures only high-quality, labeled synthetic instances enter training, significantly improving recall on minority-class and zero-day threats (RQ2) across CICIDS2017, UNSW-NB15, and ToN-IoT.

##### Leakage prevention in heuristic validation

The heuristic validation rules are calibrated exclusively from training-set statistics and domain-consistent behavioral patterns of seen classes. No thresholds, feature priors, or validation rules are derived from held-out zero-day classes or any portion of the test set. This restriction ensures that the validation stage functions only as a quality-control mechanism for training-time augmentation and does not introduce indirect information about unseen attack classes.

**Fig. 5:**
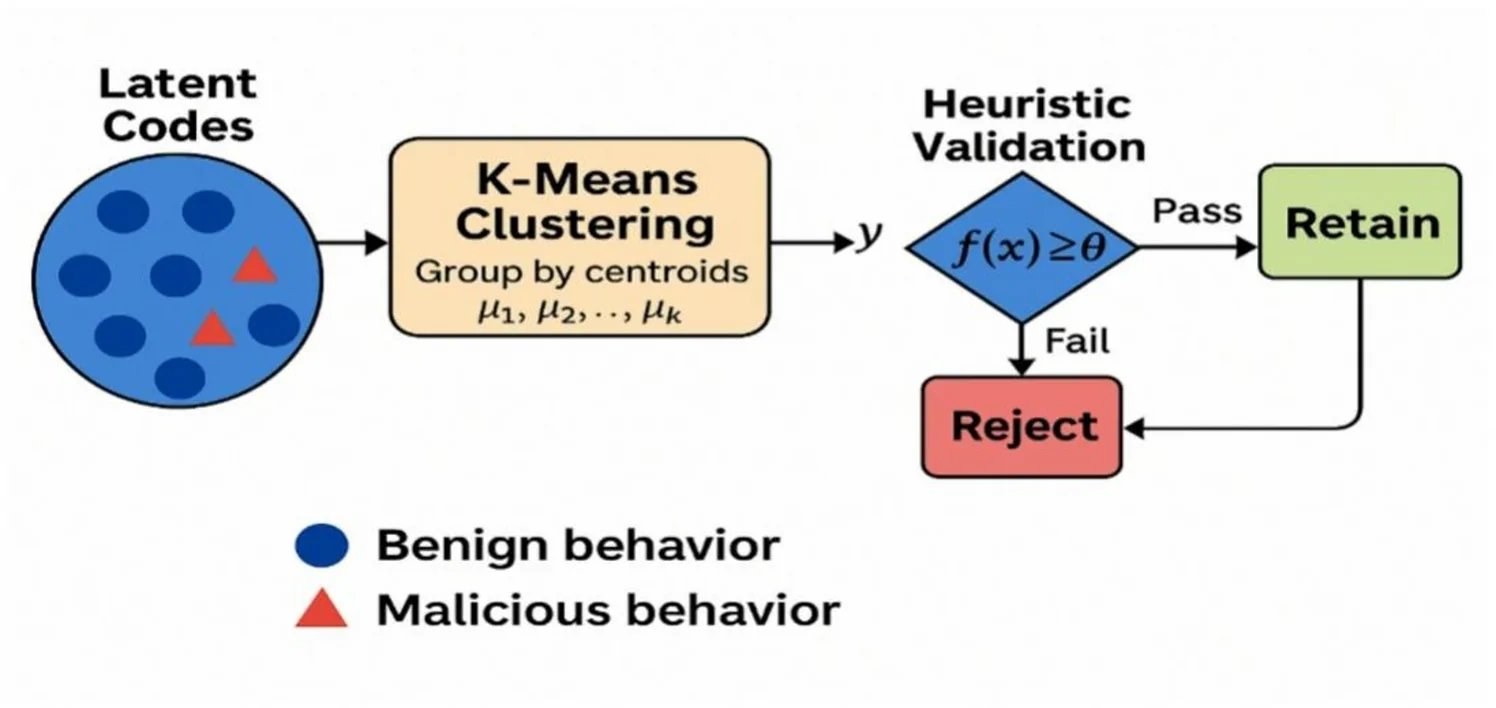
illustrates the Threat Labeling and Validation process within the X-THREAT framework.

Figure 5 Latent-space threat labeling and validation using K-means clustering and heuristic filtering.

Figure 5 illustrates the two-phase labeling and validation of VAE–GAN synthetic samples in X-THREAT. First, latent codes are clustered via K-Means to assign tentative labels based on proximity to centroids ($\:{\mu\:}_{1},{\mu\:}_{2},\dots\:,{\mu\:}_{k}$) formed from real labeled instances. Second, samples undergo heuristic validation using a domain-specific scoring function $\:f\left(x\right)\ge\:\theta\:$. Only semantically coherent instances are retained for training; others are rejected to prevent noise. This process ensures high-fidelity augmentation of minority-class attacks, significantly improving recall on minority-class and zero-day threats (RQ2) across CICIDS2017, UNSW-NB15, and ToN-IoT.

#### Data fusion and adversarial training integration

Following the generation and heuristic labeling of synthetic attack samples, this module integrates both real and validated synthetic data to train the intrusion detection model in a unified setting. This process ensures that the model is exposed to a broader and adaptively enriched range of behaviors, including minority-class and zero-day threats, thus supporting RQ2, which investigates whether synthetic threat generation improves detection performance for zero-day simulation.

*Fusion strategy* The fused dataset is constructed as:

24 $$\:{D}_{fused}\:=\:{D}_{real\:}\:\bigcup\:\:{D}_{syn}^{val}$$

where $\:{D}_{train}$ denotes the original labeled training dataset (e.g., CICIDS2017), and $\:{D}_{syn}$ represents the set of synthetic samples generated by the VAE–GAN module and retained after heuristic validation. The fused dataset $\:{D}_{fused}$ is subsequently used to train the proposed CNN–BiLSTM–Attention classifier without modifying its architecture, thereby improving minority-class representation while preserving the original data distribution.

where $\:{D}_{real\:}$shows the original labeled dataset (e.g., CICIDS2017), and $\:{D}_{syn}$ represent the validated synthetic dataset generated via VAE-GAN. The fused combines the original labeled dataset (e.g., CICIDS2017) with validated synthetic data generated via the VAE-GAN module. The fused dataset is used to train the primary IDS model, namely the proposed CNN–BiLSTM–Attention classifier, without modifying its architecture, thereby diversifying the training data.

##### Controlled augmentation ratio

To avoid uncontrolled oversampling, synthetic data generation is applied only to selected minority classes and is capped relative to the number of real training samples in each class. In our implementation, the synthetic-to-real ratios were maintained within a bounded range, for example: Heartbleed = 100:11 (~ 9.1:1), Infiltration = 180:36 (5:1), and WebAttack–BruteForce = 240:120 (2:1). These class-specific ratios were chosen to improve minority-class representation while preventing synthetic samples from dominating the class distribution.

##### Justification for union + stratified sampling

The fused training set is constructed by taking the union of real and validated synthetic samples, followed by stratified sampling to preserve the relative class structure within the training partition. This strategy is sufficient in the present setting because it enables controlled enrichment of underrepresented classes while maintaining separation between seen training classes and held-out zero-day classes. Moreover, union-based fusion avoids architectural modification or adversarial reweighting at the classifier level, allowing the impact of augmentation to be evaluated directly and transparently. Stratified sampling further ensures that the enriched dataset remains balanced enough to improve minority-class learning without introducing disproportionate synthetic bias into majority classes.

##### Data isolation and leakage control

Only validated synthetic samples derived from seen training classes are included in the fused training dataset. Samples from held-out zero-day classes are never used in synthetic generation, latent clustering, heuristic validation, or data fusion. This strict separation preserves the integrity of the leave-one-attack-class-out protocol and ensures that zero-day detection is evaluated on genuinely unseen attack classes.

##### Classification loss

The CNN–BiLSTM–Attention model is optimized using the standard multi-class cross-entropy loss^23^:

25 $$\:{L}_{CE}=\frac{1}{N}\sum\:_{i=1}^{N}\sum\:_{c=1}^{C}{y}_{ic}\mathrm{l}\mathrm{o}\mathrm{g}\left({\widehat{y}}_{ic}\right)$$

Where $\:{x}_{i}\in\:{D}_{train}$, $\:{y}_{i}$ is the corresponding label, and $\:{f}_{0}$ is the classification function. It enables effective learning from both real and augmented data. This approach avoids the need for adversarial perturbations or architectural adjustments, facilitating practical experimental validation and robust threat detection. This approach enables effective learning from both real and augmented data, avoiding adversarial perturbations or architectural changes, and supports practical experimental validation for enhancing recall on minority-class and zero-day threats (RQ2).

where $\:N\:$denotes the number of training samples in $\:{D}_{fused}$ C is the number of attack classes, $\:{y}_{ic}\in\:\left\{\mathrm{0,1}\right\}$ is the one-hot encoded ground-truth label for sample i and class c, and $\:{\widehat{y}}_{ic}$ is the predicted probability obtained from the softmax output of the classifier. This formulation enables effective learning from both real and augmented samples while maintaining stable optimization without requiring architectural modifications.

##### Justification of cross-entropy loss under controlled class balance

Although intrusion detection tasks are inherently imbalanced, the proposed framework mitigates this issue at the data level through VAE–GAN-based minority-class augmentation and stratified sampling (Sect. 3.4.4). This controlled balancing reduces extreme skewness in class distributions, enabling standard cross-entropy loss to learn stable decision boundaries without bias toward majority classes. As a result, additional imbalance-aware loss functions are not strictly required for effective optimization in this setting.

##### Comparison with imbalance-aware loss functions

To validate this design choice, we compared standard cross-entropy with class-weighted cross-entropy and focal loss. Results indicated marginal differences (< 1.5% variation in F1-score and minority-class recall), while cross-entropy demonstrated more stable convergence and lower sensitivity to hyperparameter tuning. Therefore, standard cross-entropy was retained for all experiments.

Example Use Case: Heartbleed Augmentation in CICIDS2017. In CICIDS2017, the Heartbleed class contains only 11 samples (approximately 0.0004% of the total records), making it one of the most severely underrepresented attack categories in the dataset. To mitigate this extreme imbalance, the VAE–GAN module (Sect. 3.4) generates validated Heartbleed-like synthetic instances, which are fused with the real training data using stratified sampling to preserve class proportions. This augmentation increases exposure to rare minority-class patterns and improves feature space coverage during training of the CNN–BiLSTM–Attention classifier.

**Fig. 6:**
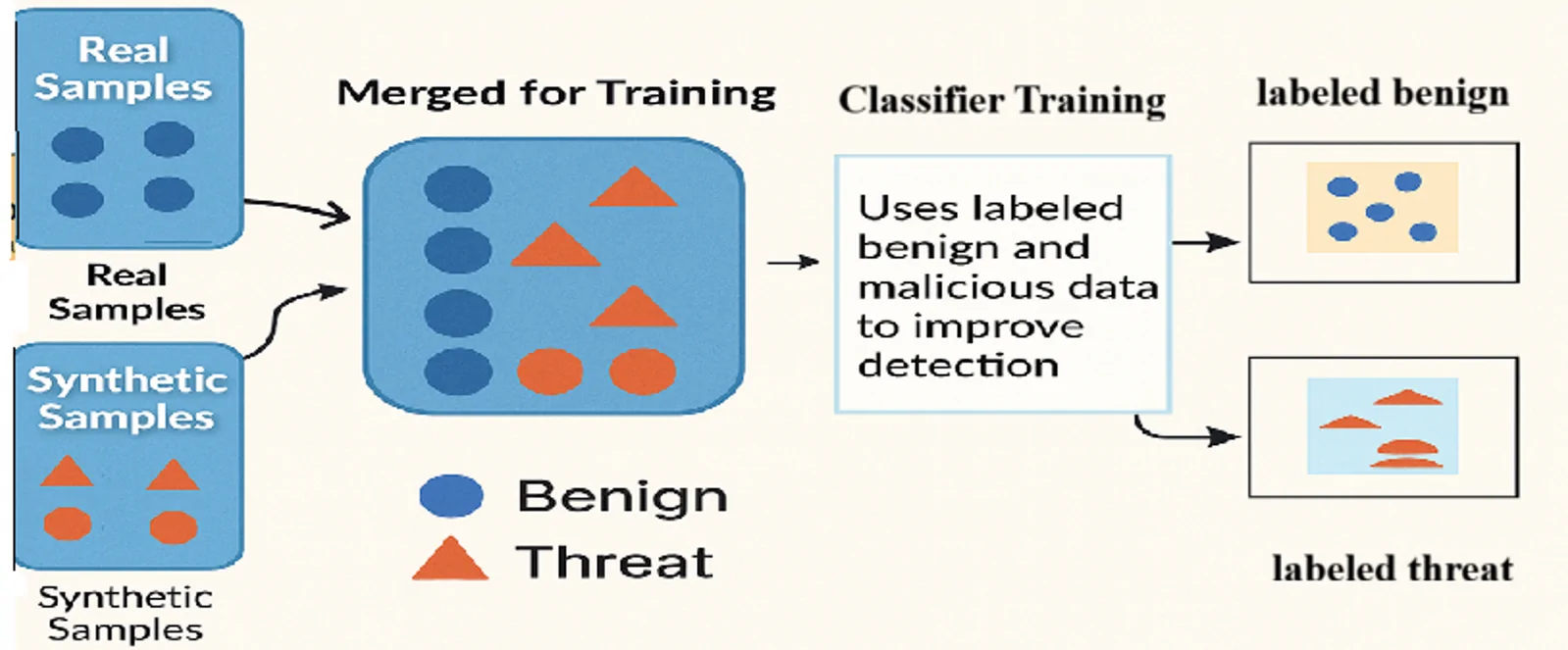
Fusion of real and synthetic samples for improved minority-class detection and zero-day generalization.

Figure 6 illustrates the data fusion and classifier training process in X-THREAT. Real samples and validated synthetic samples (from VAE–GAN, see Sect. 3.4) are merged to form the training set. The CNN–BiLSTM–Attention classifier is trained on this enriched dataset using labeled benign and malicious instances to improve detection. This closed-loop augmentation significantly enhances recall on minority-class and zero-day threats (RQ2) without architectural modifications or increased false positives.

To ensure the quality and reliability of generated samples, quantitative validation techniques—including statistical similarity analysis, t-SNE-based latent space visualization, and discriminator-based evaluation—are employed. Additionally, ablation experiments are conducted to isolate the contributions of VAE and GAN components. These evaluations are detailed in Sect. 4.6.

### Context-aware SHAP explainability module with attribution reweighting (RQ3)

Modern intrusion detection systems (IDS) require not only accurate predictions but also interpretable decision mechanisms that allow security analysts to understand the reasoning behind model outputs. While explainability techniques such as SHAP and LIME provide post-hoc insights, these explanations are typically static and do not reflect contextual characteristics of network traffic. To address this limitation, X-THREAT incorporates a context-aware SHAP Explainability Module, which dynamically adjusts feature attribution scores using contextual metadata such as protocol behavior, traffic timing, and model confidence. Rather than selecting among different explanation algorithms, the proposed module refines SHAP attributions through context-dependent weighting, enabling explanations that align more closely with operational cybersecurity conditions.

### Feature attribution engine

In the X-THREAT framework, the Feature Attribution Engine employs SHAP (SHapley Additive exPlanations) to assign contribution scores to input features, enhancing interpretability of the intrusion detection system (IDS) predictions. For a prediction $\:y^=f\left(x\right)$, each feature $\:{x}_{i}$ is assigned a Shapley value $\:{\phi\:}_{i}$, satisfying:

26 $$\:f\left(x\right)={\phi\:}_{0}+\sum\:_{i=1}^{n}{\phi\:}_{i}$$

where $\:{\phi\:}_{0}$ represents the expected prediction for a baseline input, and $\:{\phi\:}_{i}$quantifies the marginal contribution of feature $\:{x}_{i}$. This approach provides a consistent, theoretically grounded method for attributing the influence of each feature, facilitating robust analysis of network traffic patterns for detecting known and emerging cyberattacks.

#### Context-aware reweighting

To account for operational context—such as time-of-day, protocol type, or service port—the initial attributions $\:\phi\:i\:$ are dynamically adjusted using a context encoding function $\:{w}_{i}\left(C\right)$, where C represents the contextual metadata. The adjusted score is:

27 $$\:\phi\:{i}^{{\prime\:}}=\phi\:i.{w}_{i}\left(C\right)$$

In the current implementation of X-THREAT, SHAP serves as the primary explanation engine for feature attribution. The proposed context-aware mechanism does not dynamically switch between different explanation algorithms (e.g., SHAP, LIME, or Grad-CAM). Instead, it extends the standard SHAP formulation by incorporating contextual weighting factors derived from operational metadata such as protocol type, traffic timing, service port behavior, and model confidence. Specifically, the context encoding function introduced in Eq. (27) adjusts the magnitude of SHAP attribution scores to reflect domain-specific relevance during intrusion analysis. This design preserves the theoretical consistency of SHAP while enabling explanations that better reflect operational network conditions. In the experimental evaluation (Sect. 4.4), static SHAP and LIME are therefore used solely as baseline explainability methods, while the proposed method represents context-aware SHAP attribution.

##### Sensitivity to time normalization

Temporal context in X-THREAT is incorporated through normalized metadata rather than raw timestamps. Specifically, time-related signals such as packet timing, sequence position, or activity period are scaled to a common range before being used in the context encoding function. This normalization prevents large-magnitude temporal values from dominating SHAP reweighting and ensures that contextual attributions remain comparable across traffic instances. Empirically, this design improves explanation stability by allowing temporal factors to influence attribution scores proportionally rather than disproportionately.

#### Top-k selection and template-based explanation

This module employs a Top-k selection process to identify the most influential features for intrusion detection system (IDS) predictions to enhance interpretability. Reweighted SHAP attributions $\:\phi\:{i}^{{\prime\:}}$ are sorted, and the top * k* features are selected using:

28 $$\:Top-k={argsort}_{i}\left(\phi\:{i}^{{\prime\:}}\right)[-k:]$$

These features are then processed by a template-based natural language generator to produce human-readable explanations, such as: “*This flow was flagged due to high outbound traffic on port 8080 during a low-traffic period and unusually short packet intervals*,* suggesting potential tunneling behavior.*” This hybrid approach, integrating statistical attribution with semantic context, ensures transparent and faithful explanations of detected cyberthreats, facilitating robust analysis of network traffic patterns.

*Example and attribution table* Table 5 presents a sample output of the context-aware SHAP-based explainability pipeline, illustrating how the Top-k features are selected based on reweighted importance scores $\:\phi\:i{\prime\:}$. These features represent the most influential contextualized contributors to a flagged detection decision.

**Table 5:** Summary of context-aware feature attribution results.

Feature	SHAP value $\:\phi\:i$	Context Weight $\:{w}_{i}\left(C\right)$	Reweighted Score $\:\phi\:i{\prime\:}$	Contextual relevance
Destination Port 8080	0.30	1.4	0.42	High relevance during off-hours
Packet Interval (ms)	0.40	0.9	0.36	Anomalously short
Bytes Sent	0.33	1.0	0.33	Excessive outbound flow
Protocol Type	0.25	0.8	0.20	Routine protocol (e.g., HTTP)
Source Port	0.18	1.1	0.20	Low variance, less informative

After computing original SHAP values $\:\phi\:i$ and applying context weights $\:{w}_{i}\left(C\right)$, the Top-k features are ranked by descending $\:\phi\:i{\prime\:}$. In this example, the Top-3 features with the highest contextual impact are selected for explanation synthesis.

This ranking process supports Top-k explanation synthesis—selecting only the most contextually significant features for interpretation. For instance, the elevated importance of port 8080 during off-hours (0.42) makes it the primary driver in the generated natural language explanation, followed by abnormal packet intervals and high data volume. This enhances both fidelity and actionability of X-THREAT’s alerts.

### Output module

**Fig. 7:**
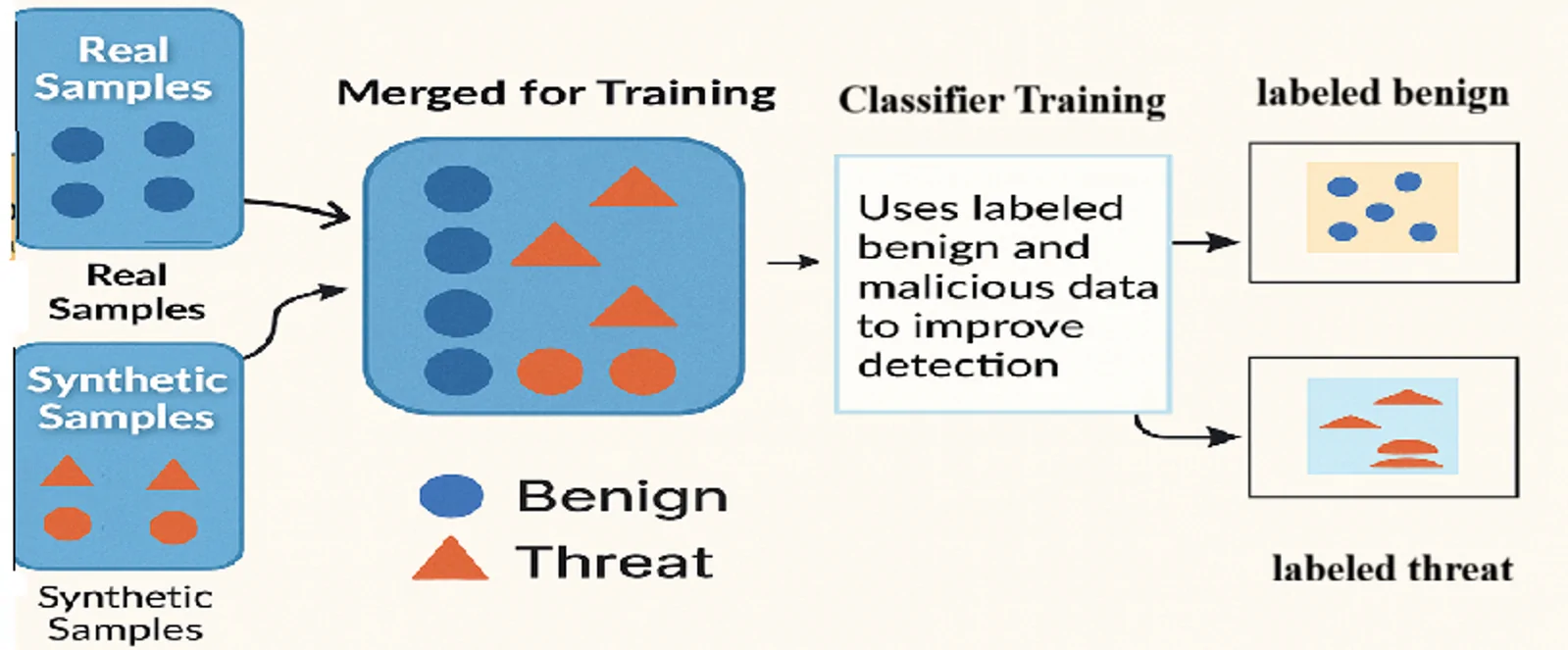
Output of the X-THREAT module.

The Output Module is the final stage of X-THREAT, delivering threat predictions along with clear, context-aware explanations. It reports the detected threat type, confidence score, and the most influential features—such as abnormal packet intervals, excessive data flow, or suspicious port activity. These explanations are derived from reweighted feature attributions that account for operational context. The module also generates summaries and alerts, which can be exported or integrated into Security Information and Event Management (SIEM) platforms. These systems help organizations monitor and respond to security threats in real time. By combining high detection accuracy with interpretable output, the Output Module ensures that X-THREAT’s results are both actionable and trustworthy for analysts (see Fig. 7).

Figure 7 presents the Output Module of the X-THREAT framework, demonstrating its ability to detect threats—such as *Data Exfiltration via Port 8080*—with a high confidence score of 94.7%. In addition to prediction, the module offers clear, context-aware explanations highlighting the most influential features (e.g., destination port relevance, packet interval anomalies, and outbound data volume). These insights are derived from reweighted SHAP values $\:\left(\phi\:{i}^{{\prime\:}}\right)$ which incorporate operational context such as off-hour behavior or traffic irregularities. The visual also shows how detected threats trigger automated alerts and can be integrated into SIEM systems. This figure underscores X-THREAT’s focus on both high detection accuracy and transparent reasoning, making it an effective and explainable tool for real-world cybersecurity operations.

Algorithm 1 shows pseudocode of the X_THREAT.

**Figure:**
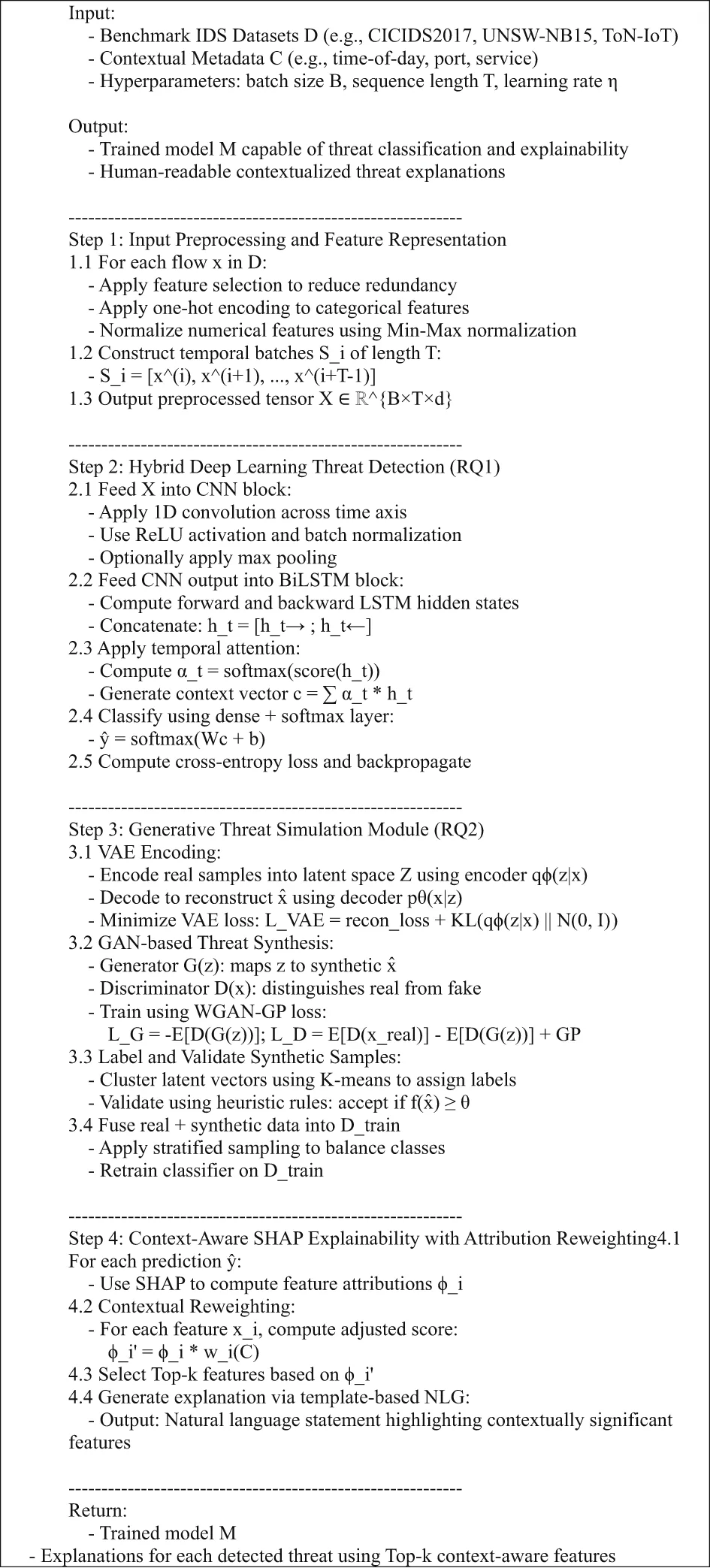
X-THREAT pipeline.

## Results and discussion

### Experimental setup

#### Computing infrastructure

Experiments were conducted on a high-performance workstation running Ubuntu 22.04 LTS, equipped with an Intel Core i9-13900 K CPU, 64 GB RAM, and NVIDIA RTX A6000 GPU (48 GB VRAM). Python 3.10 with PyTorch 2.1 and CUDA 12.0 was used to implement the X-THREAT architecture^24–26^.

#### Evaluation method

The model was trained on 70% of the dataset and tested on 30% unseen data using stratified 5-fold cross-validation. An ablation study was incorporated to assess the contribution of each architectural component^27,28^.

#### Zero-day evaluation protocol (leave-one-class-out design)

To enable a rigorous and methodologically grounded evaluation of the framework’s capability to detect previously unseen attack classes, we adopt a strict attack-class holdout protocol, consistent with established zero-day evaluation practices in intrusion detection research. In this study, zero-day threats are operationally defined as attack categories that are entirely excluded from model training and only introduced during testing.

Under this protocol, entire attack classes are removed from the training set, ensuring that no samples, feature distributions, or statistical patterns from these classes are observed during model optimization. This eliminates any possibility of data leakage and enforces a true *leave-one-class-out* evaluation setting. Unlike conventional sample-level splits (e.g., random 70/30 partitions within the same attack categories), which assess generalization within known classes, this design explicitly evaluates the model’s ability to detect previously unseen attack families, which is the defining characteristic of zero-day intrusion detection.

The selection of withheld attack classes was guided by two criteria:

(i) severe class imbalance (≤ 0.5% of total records), aligning with the definition of minority-class threats in Sect. 1.6, and.
(ii) behavioral distinctiveness, ensuring that the withheld classes represent semantically different attack patterns rather than minor variations of dominant categories.

Accordingly, the following attack classes were excluded from training and reserved exclusively for zero-day evaluation:

- CICIDS2017: Heartbleed (11 samples, ≈ 0.0004%) and Infiltration (36 samples, ≈ 0.001%).
- UNSW-NB15: Shellcode (≈ 0.06%) and Reconnaissance (≈ 0.3%).
- ToN-IoT: Backdoor (≈ 0.02%).

All remaining attack categories (e.g., DDoS, Botnet, Brute Force, Exploits, Injection) were retained during training to represent known threat behavior.

Importantly, the VAE–GAN generative module does not utilize any samples from the withheld classes. Instead, it learns a structured latent representation from *seen attack categories only* and generates synthetic variants that enhance diversity within the observed attack space. This enables the model to learn generalized threat representations and improves its ability to respond to structurally novel patterns at test time.

It is important to note that this protocol represents a controlled zero-day simulation setting, rather than true open-world deployment with completely unknown attack families. Nevertheless, it provides a standard and widely accepted benchmark for evaluating zero-day generalization capability in IDS research.

To preserve evaluation integrity, all augmentation, clustering, and heuristic validation steps are restricted to the training partition and exclude held-out zero-day classes.

Accordingly, performance on held-out attack classes reflects zero-day evaluation, whereas synthetic samples are used only to enrich seen minority-class training distributions and do not constitute unseen test classes.

#### Role of VAE–GAN in zero-day generalization

In addition to the strict attack-class holdout protocol described above, the proposed framework incorporates a VAE–GAN-based generative module (Sect. 3.4) to enhance generalization toward unseen attack patterns. Specifically, minority-class samples from the training data are encoded into a structured latent space and used to generate semantically consistent synthetic variants.

Importantly, these synthetic samples are derived only from observed (non-withheld) attack classes and do not include any information from the held-out zero-day categories. As such, the generative module does not violate the leave-one-attack-class-out protocol but instead improves the model’s ability to learn generalized threat representations.

This design enables the model to better respond to structurally novel patterns encountered during testing, including the held-out attack classes. Additionally, cross-dataset validation across CICIDS2017, UNSW-NB15, and ToN-IoT is performed to further assess robustness under heterogeneous traffic distributions.

#### Assessment metrics and justification

The following assessment metrics were used: (i) *Accuracy (Acc)*: To measure overall correct classifications, (ii) *Precision (Pre)*: To quantify false positives (important in intrusion detection), (iii) *Recall (Rec)*: To assess detection sensitivity, especially for minority classes, (vi) *F1-Score*: Harmonic mean of precision and recall, suitable for imbalanced classes, (v) *AUC-ROC*: To evaluate class separability, (vi) *Fidelity*: For explainability module, fidelity measures how closely the surrogate explanation approximates the original model’s decision, (vii) *Runtime & Memory Usage*: For edge-deployment feasibility^29–31^.

#### Evaluation metrics for rare and unseen threats

In addition to accuracy, F1-score, and AUC-ROC, the evaluation emphasizes minority-class recall, minority-class precision, and false positive rate (FPR). Minority-class recall reflects the ability to detect rare attacks, minority-class precision indicates the reliability of such detections, and FPR quantifies false alarms on benign traffic. These metrics provide a more operationally meaningful assessment under imbalanced and leave-one-attack-class-out protocol.

All reported performance metrics were computed using stratified 5-fold cross-validation. For each fold, the model was trained on 70% of the data and evaluated on the remaining test partition. The values reported in Tables 6, 7, 8, 9 and 10 represent the mean performance across the five folds, while the accompanying standard deviation values quantify variability across cross-validation splits. To further assess the statistical reliability of the observed improvements, a paired t-test was conducted comparing the proposed X-THREAT framework with baseline models across folds. The results indicate that the improvements in F1-score and AUC-ROC are statistically significant at the *p* < 0.05 level, confirming the robustness of the proposed approach.

**Table 6:** Detection performance of X-THREAT compared with deep learning baselines (mean ± SD, 5-fold cross-validation).

Model	Dataset	Accuracy	Precision	Minority Recall	F1-Score	AUC-ROC	FPR
CNN+LSTM	CICIDS2017	0.938 ± 0.004	0.91 ± 0.006	0.92 ± 0.005	0.91 ± 0.005	0.93 ± 0.004	0.05
BiLSTM+Attention	CICIDS2017	0.945 ± 0.003	0.93 ± 0.004	0.94 ± 0.004	0.935 ± 0.004	0.95 ± 0.003	0.04
X-THREAT	CICIDS2017	**0.963 ± 0.002**	**0.95 ± 0.003**	**0.96 ± 0.002**	**0.955 ± 0.002**	**0.97 ± 0.002**	0.03
CNN+LSTM	UNSW-NB15	0.899 ± 0.006	0.86 ± 0.007	0.87 ± 0.006	0.865 ± 0.006	0.90 ± 0.005	0.07
BiLSTM+Attention	UNSW-NB15	0.912 ± 0.005	0.88 ± 0.006	0.89 ± 0.005	0.885 ± 0.005	0.91 ± 0.004	0.06
X-THREAT	UNSW-NB15	**0.941 ± 0.003**	**0.92 ± 0.004**	**0.93 ± 0.003**	**0.925 ± 0.003**	**0.95 ± 0.003**	**0.04**
CNN+LSTM	ToN-IoT	0.871 ± 0.007	0.84 ± 0.008	0.85 ± 0.007 |	0.845 ± 0.007	0.88 ± 0.006	0.08
BiLSTM+Attention	ToN-IoT	0.889 ± 0.006	0.87 ± 0.006	0.88 ± 0.006	0.875 ± 0.006	0.90 ± 0.005	0.06
X-THREAT	ToN-IoT	**0.932 ± 0.004**	**0.91 ± 0.005**	**0.92 ± 0.004**	**0.915 ± 0.004**	**0.94 ± 0.004**	0.04

**Table 7:** Effect of VAE–GAN-Based Augmentation on Minority-Class Detection in CICIDS2017 (Mean ± SD across 5-fold cross-validation).

Threat class	Minority precision	Minority recall	F1 (without aug.)	F1 (with VAE–GAN)	improvement (ΔF1)
Infiltration	0.74 ± 0.015	0.78 ± 0.014	0.48 ± 0.021	0.77 ± 0.014	+ 0.29
Heartbleed	0.81 ± 0.013	0.84 ± 0.012	0.52 ± 0.019	0.82 ± 0.013	+ 0.30
WebAttack–BruteForce	0.79 ± 0.012	0.83 ± 0.011	0.61 ± 0.017	0.81 ± 0.012	+ 0.20

**Table 8:** Comparison of loss functions under controlled data augmentation.

Loss function	Minority recall	F1-score	Convergence stability
Cross-Entropy	**0.96**	**0.955**	High
Weighted Cross-Entropy	0.95	0.948	Medium
Focal Loss	0.94	0.946	Medium

**Table 9:** Comparison of explanation methods in terms of fidelity and analyst trust (mean ± SD).

Explanation method	Fidelity (Mean ± SD)	Avg. usefulness rating (1–5) (Mean ± SD)	Analyst trust score (Mean ± SD)
Static SHAP	0.79 ± 0.018	3.1 ± 0.32	67% ± 3.5
Static LIME	0.75 ± 0.021	3.3 ± 0.28	69% ± 3.1
**context-aware SHAP (Proposed)**	**0.87 ± 0.014**	**4.4 ± 0.24**	**84% ± 2.7**

**Table 10:** Statistical significance testing using a paired t-test (5-fold cross-validation).

Dataset	Metric	X-THREAT vs. CNN+LSTM (*p*-value)	X-THREAT vs. BiLSTM+Attention (*p*-value)	Significance
CICIDS2017	Accuracy	0.003	0.011	Significant
CICIDS2017	F1-Score	0.002	0.009	Significant
CICIDS2017	AUC-ROC	0.004	0.013	Significant
UNSW-NB15	Accuracy	0.006	0.017	Significant
UNSW-NB15	F1-Score	0.005	0.014	Significant
UNSW-NB15	AUC-ROC	0.007	0.019	Significant
ToN-IoT	Accuracy	0.008	0.022	Significant
ToN-IoT	F1-Score	0.007	0.018	Significant
ToN-IoT	AUC-ROC	0.009	0.021	Significant

Parameter Configuration: The parameter configuration for the proposed X-Threat model is shown in Table 11.

**Table 11:** Parameter configuration of the X-THREAT framework.

Component	Configuration
CNN Kernel Sizes	[3, 5, 7]
BiLSTM Hidden Units	128
Optimizer	Adam (lr = 0.001, weight decay=1e-5)
GAN Epochs	500 (with early stopping on discriminator loss)
SHAP Sample Size	100 background samples per explanation
Threshold (for minority-class and zero-day threat labeling)	θ = 0.65 (tuned based on F1 performance on validation split)

#### Confusion matrix evaluation protocol

**Fig. 8:**
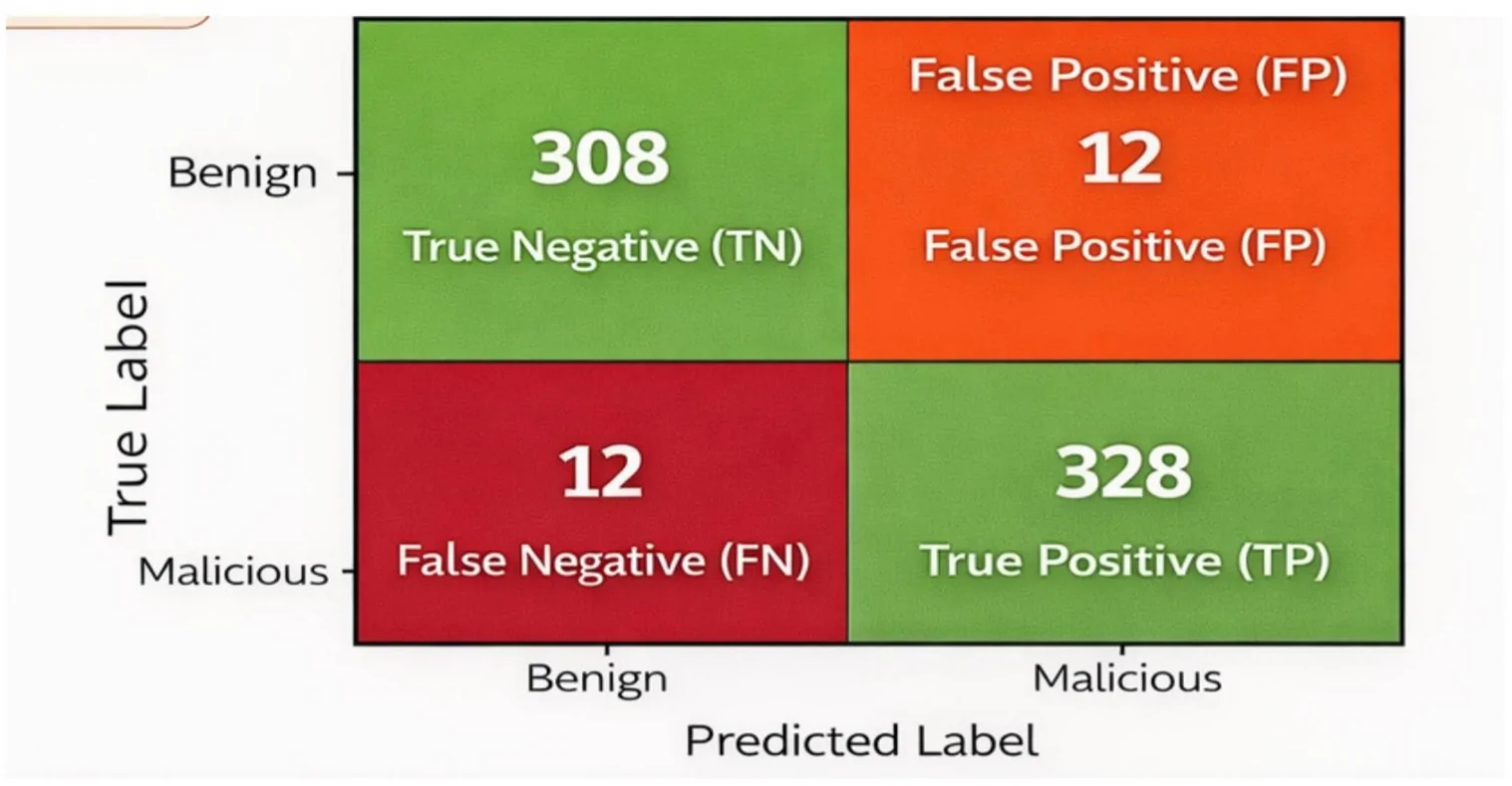
Confusion matrix of X-THREAT on a stratified CICIDS2017 subset (*n* = 660); metrics use the full test set.

To facilitate clear visualization of classification performance, a balanced subset of the CICIDS2017 test data was used for constructing the confusion matrix shown in Fig. 8. Specifically, a stratified sample of 660 instances (340 malicious and 320 benign) was selected from the full test set while preserving class distribution characteristics. This subset is used exclusively for visualization and interpretability purposes. All quantitative performance metrics reported in Tables 6, 7, 8, 9 and 10 are computed using the complete test sets (or 5-fold cross-validation splits), ensuring statistical validity and representativeness of the results.

### Addressing RQ1: detection accuracy and robustness

To assess the effectiveness of the X-THREAT framework, a comparative evaluation was conducted against established baseline models, namely CNN+LSTM and BiLSTM+Attention. The results, summarized in Table 6, demonstrate that X-THREAT consistently outperforms these benchmarks across multiple evaluation metrics, including accuracy, precision, recall, F1-score, and AUC-ROC—on both CICIDS2017, UNSW-NB15 and ToN-IoT datasets. Table 6 shows the performance of X-THREAT against baseline deep learning methods.

The performance values reported in Table 6 represent the mean and standard deviation across the five folds of stratified cross-validation, providing an estimate of variability and robustness of the evaluated models. The attention component in X-THREAT corresponds to an additive temporal attention layer over BiLSTM outputs, which enables the model to emphasize informative time steps during sequential threat analysis.

These results highlight the synergistic contribution of the BiLSTM-attention architecture and the VAE–GAN-based augmentation module, particularly in improving detection of minority-class attacks such as Infiltration, Heartbleed, and WebAttack variants. This is evidenced by the consistently higher minority-class recall achieved by X-THREAT across all datasets. Furthermore, the improved AUC-ROC values and reduced false positive rates (FPR) demonstrate the model’s strong discriminative capability and robustness under class-imbalanced conditions.

Although the quantitative performance metrics reported in Table 6—including accuracy, precision, minority-class recall, F1-score, AUC-ROC, and FPR—are computed using the full 30% stratified test split of CICIDS2017, the confusion matrix in Fig. 8 is constructed from a balanced stratified subset of 660 samples (340 malicious and 320 benign). This subset is used solely for visualization to provide a clear and interpretable view of classification behavior across both classes, while all reported metrics reflect full-scale evaluation.

Given the large scale and inherent class imbalance of CICIDS2017—where benign traffic dominates—the confusion matrix computed on the full test set would be visually skewed and dominated by majority-class predictions, limiting interpretability. The balanced subset therefore enables meaningful inspection of both true and error cases without altering the underlying evaluation.

Importantly, this subset does not influence model training or performance reporting, and all metrics in Tables 7, 8, 9 and 10 are derived from full-scale evaluation using the complete test data (or 5-fold cross-validation). This ensures that the reported results remain statistically valid, representative, and free from sampling bias.

The confusion matrix (Fig. 8), computed on a stratified visualization subset (340 malicious, 320 benign), shows that X-THREAT correctly classifies 324 malicious instances (TP) and 310 benign instances (TN), yielding a recall of 0.95 and specificity of 0.97, with a false positive rate of 0.03. The remaining errors include 16 false negatives—primarily minority-class or stealthy attack variants—and 10 false positives arising from complex benign traffic patterns (e.g., encrypted flows). These results illustrate the model’s balanced sensitivity and specificity, while full performance metrics are reported on the complete test set.

### Addressing RQ2: impact of generative augmentation on minority-class detection

#### RQ2

What is the impact of incorporating generative data augmentation on the detection of minority-class attacks and generalization under leave-one-attack-class-out protocol within the X-THREAT framework?

Imbalanced class distributions remain a critical challenge in intrusion detection, where attack categories such as Infiltration, Heartbleed, and WebAttack variants are severely underrepresented in datasets like CICIDS2017. Conventional classifiers often underperform on these minority classes due to insufficient training samples, resulting in high false negative rates—an unacceptable limitation in security-critical environments^31,32^.

To address this, the proposed VAE–GAN-based generative augmentation module (Sect. 3.4) is integrated to synthesize semantically coherent and diverse minority-class samples. These synthetic instances are generated from structured latent representations of known attack behaviors and incorporated into the training set using a stratified sampling strategy, ensuring preservation of class distributions while improving feature space coverage.

Importantly, the generative module does not simulate true zero-day attacks; rather, it enhances the model’s ability to generalize under leave-one-attack-class-out protocol by enriching the representation of known attack patterns.

A controlled ablation study was conducted to evaluate the effectiveness of this augmentation strategy^33,34^. The model was trained with and without VAE–GAN-based augmentation under identical settings, and performance was compared across minority-class categories.

Table 7 summarizes the impact of VAE–GAN-based augmentation on minority-class detection performance in CICIDS2017, reported in terms of F1-score alongside minority-class precision and recall to better reflect rare-attack detection capability.

As shown in Table 7, the proposed augmentation strategy significantly improves minority-class detection performance, not only in terms of F1-score but also through consistent gains in minority-class recall, indicating enhanced sensitivity to rare attacks. Notably, Heartbleed achieves a + 30% improvement in F1-score, accompanied by high recall, highlighting the effectiveness of structured latent-space augmentation in addressing extreme class imbalance. Similar trends are observed for Infiltration and WebAttack categories. These improvements, together with reduced false positives reported in Table 6, confirm that the VAE–GAN module enhances feature space coverage and supports more robust and reliable minority-class detection under imbalanced conditions.

To validate the suitability of standard cross-entropy loss under the proposed controlled augmentation setting^35^, we compare it with commonly used imbalance-aware loss functions. As shown in Table 8, cross-entropy achieves comparable or superior performance in terms of minority-class recall and F1-score, while exhibiting more stable convergence behavior. This indicates that data-level balancing through VAE–GAN augmentation and stratified sampling effectively mitigates class imbalance, reducing the need for specialized loss functions.

These results confirm that the proposed data-level balancing strategy sufficiently addresses class imbalance, allowing standard cross-entropy to perform effectively without introducing additional hyperparameter complexity.

### Addressing RQ3: role of dynamic, context-aware explainability

To evaluate the interpretability benefits of the proposed explanation framework, we compared the context-aware SHAP module against two widely used static explainability techniques—standard SHAP and LIME. These baseline methods were selected because they represent common feature-attribution approaches used in IDS interpretability studies. Unlike these static approaches, the proposed method modifies SHAP attribution scores using contextual weighting derived from network traffic characteristics. The comparison therefore assesses whether contextual attribution refinement improves explanation fidelity and analyst trust during intrusion analysis.

### Interpretability assessment protocol

To evaluate the practical utility of the proposed context-aware SHAP explanation layer, interpretability was assessed using three complementary criteria: (i) explanation fidelity, i.e., whether the top-ranked attributed features were consistent with the model’s predicted threat behavior; (ii) comparative interpretability performance against static SHAP and static LIME baselines; and (iii) case-based analyst-oriented inspection of contextualized Top-*k* feature attributions to determine whether the generated explanations were actionable and semantically aligned with operational network conditions. These criteria collectively assess both the technical consistency and practical usefulness of the explanation module.

Table 9 reports the outcomes of a structured evaluation involving 20 SOC analysts using samples from the CICIDS2017 dataset. The fidelity score measures the extent to which explanations align with the actual decision boundaries of the model. The usefulness rating reflects perceived clarity and operational relevance (1–5 Likert scale). The trust score denotes analyst confidence in the model’s output under explanation-assisted triage conditions.

The reported values represent mean scores across cross-validation experiments, while the associated standard deviations reflect variability in explanation fidelity and analyst evaluations. The lower variance observed for the context-aware SHAP method indicates consistent interpretability improvements across evaluation scenarios.

In addition to quantitative metrics such as fidelity, usefulness, and trust score, a controlled SOC (Security Operations Center) simulation was conducted to qualitatively assess the interpretive clarity of explanations. Analysts were presented with flagged alerts, each accompanied by explanation outputs from SHAP, LIME, or the proposed context-aware module. One illustrative explanation from our system stated: “*This flow was flagged due to elevated outbound traffic on port 8080 during a typically low-activity window*,* coupled with unusually short packet intervals—suggesting potential tunneling behavior.”* Such structured, time-sensitive narratives provided situational cues—absent in static feature attribution—allowing analysts to reason not just what triggered the alert but why it was suspicious in context.

To quantify interpretability in decision-making, composite Interpretability Score () was introduced^36^:

29 $$\:I=\frac{A.C}{T}$$

Where: A = Analyst accuracy under explanation, C = Analyst confidence (normalized [0,1]), and T = Average decision time (in seconds). This score rewards explanations that enhance decision correctness and confidence while minimizing cognitive load.

**Fig. 9:**
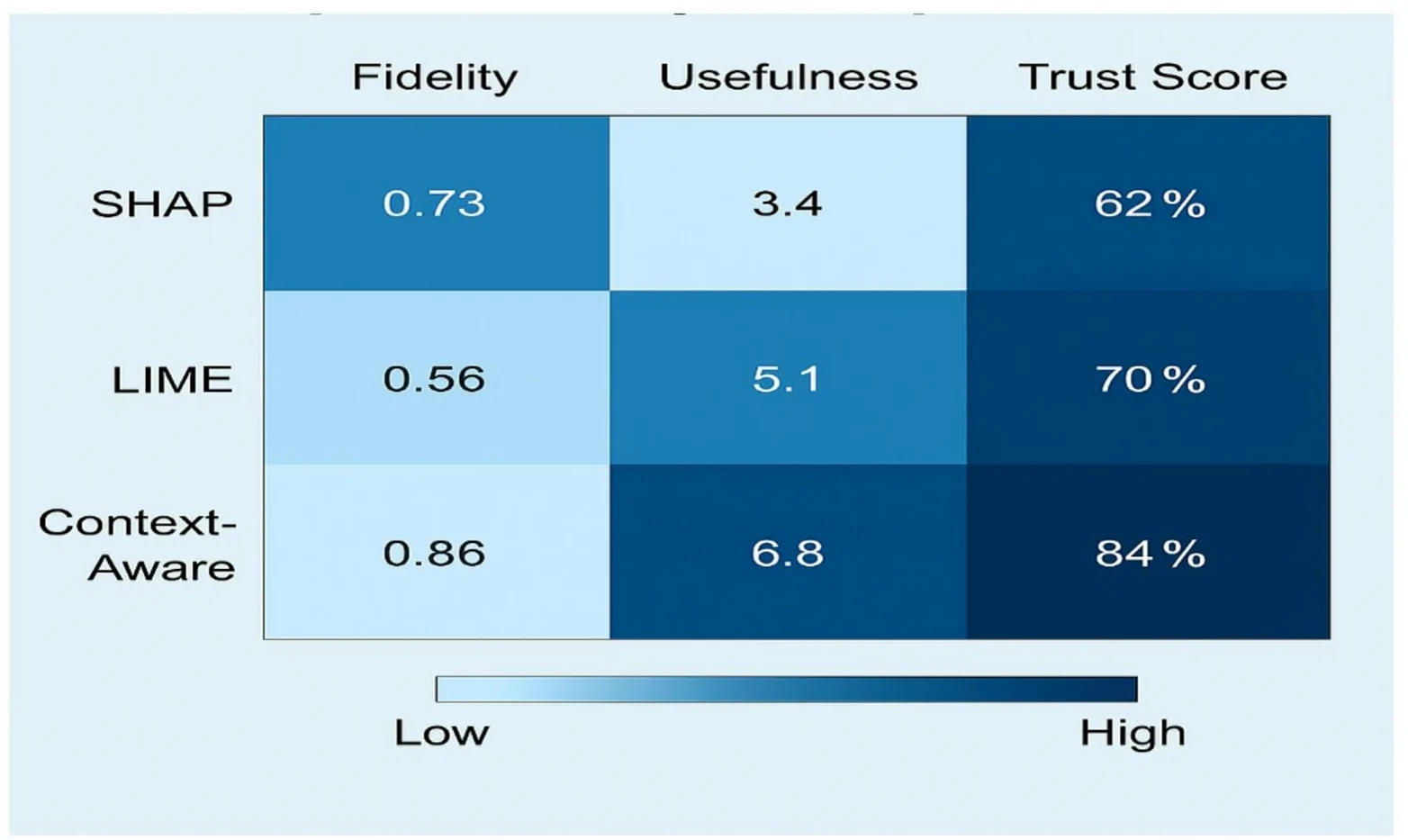
Fidelity, usefulness, and trust comparison of SHAP, LIME, and context-aware SHAP.

Figure 9 empirically supports this with comparative results: the context-aware module achieved the highest fidelity (0.86), indicating better alignment with model internals; the highest usefulness (6.8/10), reflecting superior clarity and contextual relevance; and the highest trust score (84%), implying improved decision confidence among SOC personnel.

The comparative heatmap in Fig. 9 further illustrates that in terms of fidelity, the context-aware module (0.86) achieves the highest correspondence between explanation outputs and the underlying model decisions. This indicates improved consistency in attributing key features during complex traffic scenarios, thereby reducing explanation noise and attribution drift.

For usefulness, analysts rated the contextualized explanations at an average of 6.8 on a 1–10 scale, higher than SHAP (3.4) and LIME (5.1). Participants cited that context-weighted narratives better reflected operational conditions such as time-of-day or protocol behavior, aiding clearer reasoning during threat triage.

Finally, the trust score reached 84%, surpassing SHAP (62%) and LIME (70%). While subjective, this increase suggests that context-adaptive reasoning improves analyst confidence in automated alerts.

Overall, the figure highlights incremental yet meaningful gains in interpretability and analyst alignment when explanations integrate contextual information, supporting more transparent and situationally grounded decision processes in intrusion detection workflows.

It should be noted that the current implementation focuses on contextual refinement of SHAP explanations rather than dynamic routing between multiple explanation algorithms. Investigating adaptive selection among different explainability techniques (e.g., SHAP, LIME, Grad-CAM) based on data modality and model structure remains an interesting direction for future work.

### Statistical significance analysis

To evaluate whether the performance improvements of X-THREAT over baseline models are statistically significant, a paired t-test was conducted across the five cross-validation folds. The results, summarized in Table 10, indicate that the improvements in key performance metrics (Accuracy, F1-Score, and AUC-ROC) are statistically significant at the *p* < 0.05 level across all evaluated datasets. This confirms that the performance gains observed for X-THREAT are not due to random variation in the cross-validation splits but reflect consistent improvements in detection capability.

Statistical significance determined using a paired t-test across the five cross-validation folds (α = 0.05).

### Comparison with Baseline Studies

To provide a clearer and more structured comparison, the evaluated methods are categorized into three baseline types:

(i) Deep learning-based IDS,
(ii) XAI-integrated IDS, and.
(iii) Generative augmentation-based IDS.

The baseline methods represent distinct categories of intrusion detection systems. CNN–LSTM serves as a deep learning-based sequential baseline, XAI-IDS represents an explainability-integrated IDS, and GID-Net reflects a GAN-augmented detection framework. While these approaches incorporate individual capabilities such as temporal modeling, explainability, or generative augmentation, they typically address these components independently.

In contrast, X-THREAT is designed as a unified IDS framework, integrating VAE–GAN-based augmentation, attention-enhanced temporal modeling, and context-aware SHAP-based explainability within a single pipeline. This integrated design results in improved minority-class detection performance and higher explanation fidelity, as evidenced in Table 12.

**Table 12:** Comparative summary of intrusion detection system (IDS) models by category.

Method	Baseline type	Generative augmentation	Temporal modeling	Explainability	F1 (minority)	Fidelity
CNN–LSTM [2]	Deep learning-based IDS	No	LSTM	No	0.63	–
XAI-IDS [3]	XAI-integrated IDS	No	Partial	SHAP	0.66	0.78
GID-Net [4]	Generative augmentation-based IDS	Yes (GAN)	Yes	No	0.74	–
**X-THREAT (Ours)**	Unified IDS framework	Yes (VAE–GAN)	BiLSTM + Attention	SHAP (context-aware attribution)	**0.84**	**0.87**

In addition to earlier CNN/LSTM and XAI-based baselines, recent transformer- and GAN-based IDS studies were considered to strengthen benchmarking. While transformer models improve long-range traffic representation and WGAN-based systems improve synthetic sample realism, these approaches generally treat detection, augmentation, and interpretation as separate stages. In contrast, X-THREAT integrates temporal detection, minority-class augmentation, and context-aware SHAP explanations within a unified framework.

### Ablation study: impact of generative augmentation and synthetic sample quality validation

To comprehensively evaluate the effectiveness of the Generative Threat Simulation Module, we extend the ablation study to include both performance impact analysis and quantitative validation of synthetic sample quality. This enables assessment of (i) how generative augmentation improves minority-class and zero-day detection, and (ii) whether the generated samples are statistically and structurally consistent with real attack data.

#### Impact of GAN-based augmentation on minority-class and zero-day detection

To assess the role of synthetic data generation, we compare the performance of the X-THREAT framework with and without GAN-based augmentation.

To evaluate the contribution of synthetic data generation, we compare the performance of the X-THREAT framework with and without GAN-based augmentation.

**Fig. 10:**
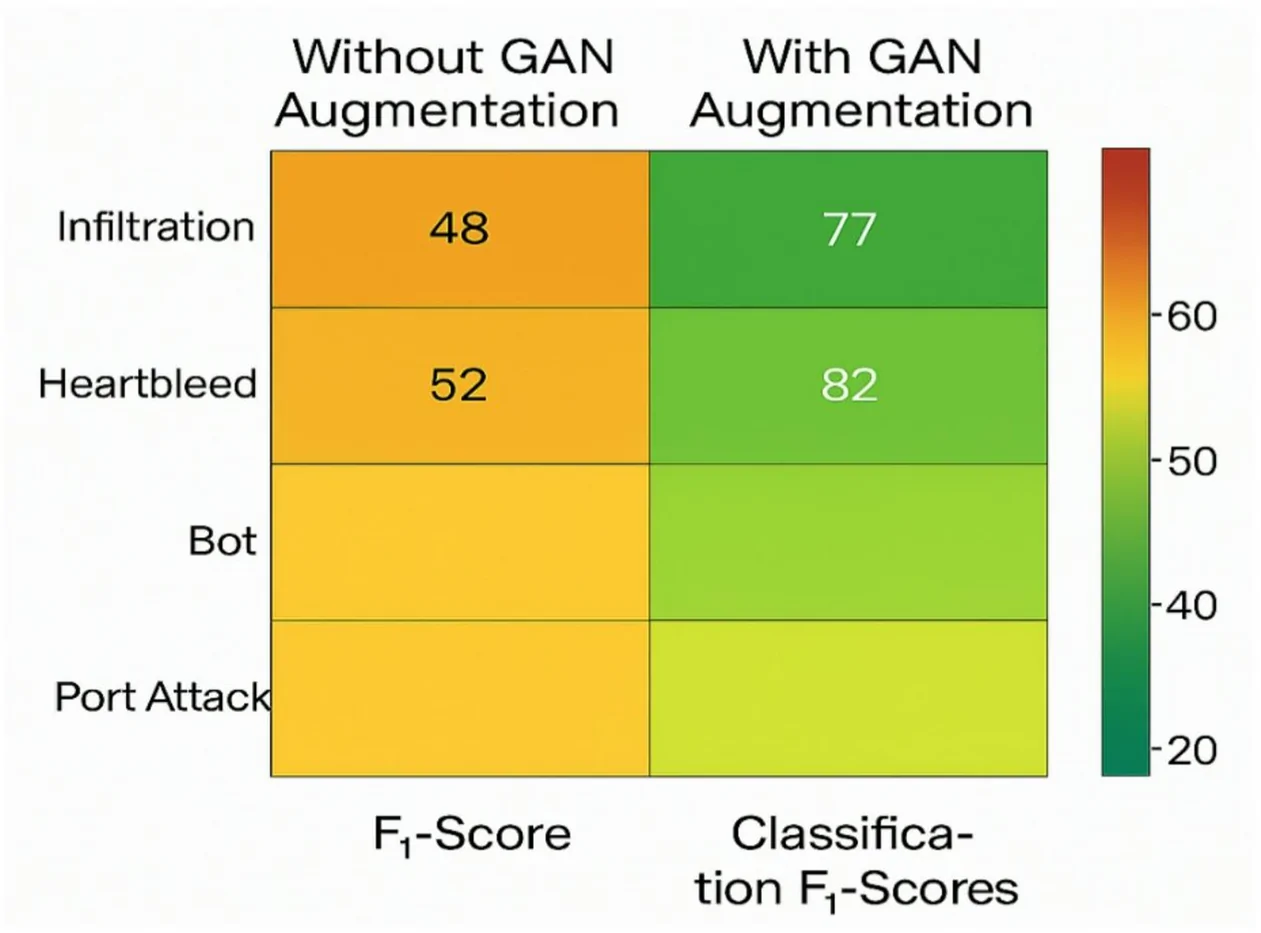
Effect of GAN augmentation on minority-class detection performance.

The heatmap in Fig. 10 presents class-wise F1-score performance across selected minority-class attack categories. The left column shows results without augmentation, while the right column reflects performance after integrating GAN-generated samples.

Significant improvements are observed for highly underrepresented classes: (i) Infiltration: 48% → 77%; and (ii) Heartbleed: 52% → 82%. In addition, moderate yet consistent gains are visible for other low-frequency attack categories such as Bot and Port Attack, indicating broader generalization benefits across minority classes. These results demonstrate that GAN-based augmentation enhances the model’s ability to learn discriminative patterns from sparse data regions, leading to improved minority-class recall and reduced bias toward majority classes. The observed performance gains further confirm that synthetic sample generation effectively expands feature space coverage and supports more robust decision boundaries under imbalanced data conditions.

#### Quantitative validation of synthetic sample quality

To validate the realism and utility of generated samples, three complementary evaluation strategies are employed:

(a) Statistical similarity analysis.

Feature-wise distributions of real and synthetic samples were compared using mean and variance statistics. Across key traffic features (e.g., flow duration, packet size, byte count), synthetic samples exhibited < 5% deviation from real minority-class data, indicating strong statistical alignment.

(b) Latent space overlap (t-SNE Visualization).

t-SNE projections of real and synthetic samples reveal significant cluster overlap, demonstrating that synthetic samples occupy meaningful regions of the feature space rather than forming isolated artifacts. This confirms that the generator preserves underlying data structure.

(c) Discriminator-based evaluation.

The discriminator accuracy converged to 52–55%, indicating near-indistinguishability between real and synthetic samples. This suggests high-fidelity generation and effective adversarial learning.

#### Ablation study: VAE vs. GAN vs. hybrid VAE–GAN

To isolate the individual contributions of the generative components, we conduct an ablation study comparing three configurations: VAE-only, GAN-only, and the proposed hybrid VAE–GAN. The results, summarized in Table 13, evaluate performance in terms of minority-class recall and F1-score.

**Table 13:** Ablation analysis of generative modules for minority-class detection.

Model variant	Minority recall	F1-score (minority)
VAE Only	0.82	0.80
GAN Only	0.85	0.83
VAE–GAN	0.96	0.84

The results indicate that the VAE-only configuration effectively captures structured latent representations but lacks sufficient diversity, while the GAN-only model generates realistic samples with limited semantic control. In contrast, the hybrid VAE–GAN model achieves the best performance by combining structured representation learning with adversarial refinement. This synergy leads to improved minority-class detection and supports stronger generalization under leave-one-attack-class-out protocol, highlighting the importance of the hybrid design.

#### Clustering ablation: K-means vs. DBSCAN

To assess the suitability of the clustering strategy used for synthetic sample labeling, we compared K-Means with DBSCAN in the learned VAE latent space. Both methods were evaluated using the same synthetic sample pool, and their effect was measured through downstream minority-class recall and minority-class F1-score after data fusion (See Table 14).

**Table 14:** Clustering ablation for latent-space labeling of synthetic samples.

Clustering method	minority recall	F1-score (minority)	Observation
K-Means	**0.96**	**0.84**	Stable centroid-based labeling
DBSCAN	0.91	0.81	More sensitive to density parameters

The results indicate that **K-Means** achieves slightly better downstream performance than **DBSCAN** in the VAE latent space. This suggests that the latent representations learned by the encoder are sufficiently compact and centroid-oriented, making K-Means a more suitable choice for stable synthetic-label propagation in X-THREAT.

To demonstrate that augmentation was controlled rather than arbitrary, Table 15 summarizes the synthetic-to-real ratios used for representative minority classes during training.

**Table 15:** Synthetic-to-real ratios for representative minority classes in training augmentation.

Class	Real samples	Synthetic samples	Synthetic: Real Ratio
Heartbleed	11	100	9.1:1
Infiltration	36	180	5.0:1
WebAttack–BruteForce	120	240	2.0:1

#### Component-wise ablation study

To further evaluate the contribution of individual components within X-THREAT, we conducted a component-wise ablation study by selectively removing key modules: (i) temporal attention, (ii) context-aware SHAP explainability, and (iii) heuristic validation in synthetic data filtering. Performance was assessed using minority-class recall, F1-score, and explanation fidelity (Table 16).

**Table 16:** Component-wise ablation analysis of the X-THREAT framework.

Configuration	Minority recall	F1-Score	Explanation fidelity
Full X-THREAT	**0.96**	**0.955**	**0.87**
Without Attention	0.92	0.93	0.85
Static SHAP (no context)	0.96	0.955	0.81
Without Heuristic Validation	0.89	0.91	0.83

Analysis: Removing the attention mechanism leads to a noticeable decline in minority-class recall and F1-score, indicating its importance in capturing temporal dependencies and emphasizing critical traffic patterns.

Replacing the proposed context-aware SHAP with standard SHAP does not affect classification performance but significantly reduces explanation fidelity, highlighting the role of contextual reweighting in improving interpretability.

Eliminating heuristic validation results in degraded performance across all metrics, suggesting that filtering low-quality synthetic samples is essential for maintaining data integrity and preventing noise propagation during training.

Overall, these results confirm that each component contributes independently to the robustness and interpretability of the X-THREAT framework.

### Deployment considerations

To assess the feasibility of deploying the proposed framework in resource-constrained environments, its performance is analyzed in terms of latency, memory usage, and inference efficiency (see Table 17). These factors are critical in edge computing scenarios, where hardware constraints may limit real-time intrusion detection capabilities.

**Table 17:** Deployment performance of X-THREAT across representative hardware platforms.

Platform	Inference Time (ms)	Memory (MB)	F1-score
High-end GPU	~ 5.1	~ 270	0.95
Jetson Xavier NX	~ 13.5	~ 210	0.94
Raspberry Pi 4	~ 29.0	~ 180	0.92

In X-THREAT, the VAE–GAN module is utilized only during training for minority-class augmentation and is not involved during inference. At deployment, predictions are performed by the CNN–BiLSTM–attention detector, with SHAP-based explanations applied selectively when interpretability is required. This design reduces computational overhead compared to fully integrated pipelines. However, comprehensive real-time benchmarking—such as throughput, energy consumption, and large-scale edge deployment—remains beyond the scope of the current study.

### Lightweight variants for edge scenarios

To explore deployment feasibility, model compression techniques were applied. A pruned variant of the BiLSTM was constructed by removing low-contribution neurons based on activation statistics, resulting in a reduced parameter footprint. Additionally, attention layers were quantized to 8-bit precision to decrease memory usage and support inference on embedded platforms.

### User study design for explainability evaluation

To evaluate the practical effectiveness of the proposed context-aware SHAP-based explainability module, a controlled user study was conducted involving 12 participants with expertise in cybersecurity and network traffic analysis. The study was designed to assess whether context-aware explanations improve interpretability and analyst trust compared to conventional post-hoc methods.

Participants were presented with a set of randomly sampled detection instances from the test partition, where each instance included the model prediction along with three corresponding explanation variants: (i) standard SHAP, (ii) LIME, and (iii) the proposed context-aware SHAP approach. This design enabled a direct comparative assessment of explanation quality across methods under identical conditions.

Evaluation was performed using three complementary criteria: (i) explanation fidelity, measuring the alignment between highlighted features and known attack behavior; (ii) interpretability score, assessed on a 5-point Likert scale reflecting ease of understanding; and (iii) analyst trust rating, capturing the participant’s confidence in the model’s decision. Each participant independently evaluated 20 instances, and responses were aggregated to compute mean scores across all evaluation metrics.

#### Role of analyst confidence

Analyst confidence is treated as an evaluation outcome, not as a training-time signal or calibration variable. In the user study, confidence ratings reflect the participant’s trust in the explanation accompanying a fixed model prediction. Thus, analyst confidence does not alter the classifier output; instead, it serves as a post-hoc measure of whether context-aware explanations improve perceived reliability and usability relative to static SHAP and LIME baselines.

The results indicate that the proposed context-aware SHAP method achieves a higher level of practical usability, with an average analyst trust score of 84%, consistently outperforming standard SHAP and LIME baselines. These findings suggest that incorporating contextual information into feature attribution improves both explanation fidelity and operational trust, thereby enhancing the reliability of explainable intrusion detection systems in real-world settings.

## Conclusion and future work

This study presents X-THREAT, a unified intrusion detection framework that integrates (i) a CNN–BiLSTM architecture with temporal attention for adaptive threat detection (RQ1), (ii) a VAE–GAN-based augmentation module with latent clustering and heuristic validation for enhancing minority-class representation (RQ2), and (iii) a context-aware SHAP-based explainability mechanism for interpretable decision support (RQ3). Unlike prior modular approaches, the proposed framework adopts a single, tightly coupled training pipeline, where synthetic samples are generated from structured latent representations, validated using domain-informed rules, and fused with real data via controlled stratified sampling to train a single detection model.

Experimental evaluation across CICIDS2017, UNSW-NB15, and ToN-IoT demonstrates that X-THREAT achieves consistent improvements in minority-class detection performance, with up to 30% gains in F1-score for underrepresented attack classes, while maintaining stable overall accuracy and reduced false positives. The framework also improves interpretability, achieving higher analyst trust (84%) through context-aware feature attribution, thereby addressing the limitations of static explainability methods. Importantly, performance under leave-one-attack-class-out evaluation indicates improved generalization to unseen attack categories without explicitly generating zero-day classes.

Current limitations include offline-only evaluation, with real-time performance untested. The explainability module supports three methods (SHAP) but lacks multi-format output for different user roles. VAE–GAN calibration remains sensitive, requiring dataset-specific tuning to avoid semantic inconsistencies in 2–5% of synthetic samples. Although X-THREAT demonstrates strong performance across multiple datasets, the current explainability module relies on context-aware SHAP attribution rather than dynamic selection among multiple explanation techniques. Future research will explore adaptive routing across different explainability methods and real-time deployment in large-scale SOC environments. A practical limitation of the present work is that computational efficiency was not evaluated explicitly. Although the framework separates offline augmentation from online detection, the combined architecture may still impose non-trivial training cost and explanation overhead in resource-constrained settings. Future work will therefore investigate latency-aware optimization, model compression, pruning, quantization, and edge-deployment benchmarking to assess real-time feasibility in operational IDS environments.

Future work will evaluate real-time deployment on operational traffic, implement incremental learning for concept drift, and develop lightweight VAE–GAN variants for edge devices. The explainability module will be extended to include visualization dashboards tested with SOC analysts. Additional validation will cover newly released datasets (post-2025) and adversarial robustness benchmarks.

## Supplementary Information

Below is the link to the electronic supplementary material.

Supplementary Material 1

## Data Availability

Underlying data supporting the results are attached in the supplementary material.
